# Adaptive Mobile Health Intervention to Reduce Excess Gestational Weight Gain: A Cluster-Randomized Clinical Trial

**DOI:** 10.1001/jamanetworkopen.2026.8007

**Published:** 2026-04-20

**Authors:** Monique M. Hedderson, Susan D. Brown, Charles P. Quesenberry, Fei Xu, Emily F. Liu, Karen L. Li, Sneha B. Sridhar, Tali Sedgwick, Page Kissel, Hillary D. Serrato Bandera, Mibhali M. Bhalala, Cheryl L. Albright, Assiamira Ferrara

**Affiliations:** 1 Kaiser Permanente Northern California, Division of Research, Pleasanton; 2 Center for Upstream Prevention of Adiposity and Diabetes Mellitus, Kaiser Permanente Northern California, Division of Research, Pleasanton; 3 Kaiser Permanente Bernard J. Tyson School of Medicine, Pasadena, California; 4 Department of Internal Medicine, University of California, Davis, Sacramento; 5 The Permanente Medical Group, Kaiser Permanente Northern California, Pleasanton; 6 University of Hawaii at Manoa School of Nursing and Dental Hygiene, Honolulu

## Abstract

**Importance:**

Excessive gestational weight gain (GWG) occurs in 50% of pregnant patients with overweight or obesity and is associated with several perinatal complications. Scalable, evidence-based lifestyle interventions are needed.

**Objective:**

To test whether an adaptive mobile health lifestyle intervention plus standard care reduces excessive GWG compared with standard care alone.

**Design, Setting, and Participants:**

This cluster-randomized clinical trial, with randomization at the clinician level, enrolled pregnant patients with a singleton pregnancy, prepregnancy body mass index (BMI; calculated as weight in kilograms divided by height in meters squared) 25.0 to less than 40.0, and receiving care within an integrated health system. Data were analyzed from May 1, 2024, to February 12, 2026.

**Interventions:**

Clinicians randomized to the intervention received motivational interviewing guidance for discussing GWG, and their patients received personalized, automated feedback on GWG and physical activity via a smartphone application; a wireless scale and activity tracker; 13 weekly education topics delivered via app; and step-wise chats and/or phone support from a lifestyle coach, for those experiencing accelerated GWG.

**Main Outcomes and Measures:**

The co–primary outcomes were weekly rate of GWG and total GWG (both continuous and categorized per IOM guidelines).

**Results:**

From a total of 58 clinicians who consented to participate, 1265 patients (mean [SD] age, 33.4 (4.6) years; mean [SD] prepregnancy BMI, 29.8 [3.8]) consented to participate and were enrolled in the trial, including 677 patients in the intervention group and 588 patients in the standard care condition. The weekly rate of GWG was significantly lower in the intervention group vs standard care group (mean [SD] rate, 0.25 [0.20] vs 0.28 [0.20] kg/week; mean between-group difference, −0.03 [95% CI, −0.05 to −0.01] kg/week). Fewer patients in the intervention group exceeded IOM guidelines for weekly rate of GWG than in the standard care group (351 patients [51.9%] vs 354 patients [60.2%]; RR, 0.86 [95% CI, 0.78-0.95]), while more patients gained below the guidelines (148 patients [21.8%] vs 101 patients [17.2%]; relative risk [RR], 1.27 [95% CI, 1.01-1.61]). Total GWG was significantly lower in the intervention group than the standard care group (mean [SD], 9.7 [6.2] vs 10.6 [6.2] kg; mean between-group difference, −0.87 [95% CI, −1.40 to −0.34] kg). Fewer patients in the intervention exceeded IOM guidelines for total GWG than in standard care (299 patients [44.1%] vs 301 patients [51.2%]; RR, 0.87 [95% CI, 0.77-0.98]) while more patients gained below the total GWG guidelines [182 patients [26.8%] vs 126 patients [21.5%]; RR, 1.23 [95% CI, 1.04-1.45]).

**Conclusions and Relevance:**

This cluster-randomized trial found that an adaptive intervention leveraging technology effectively reduced the rate of GWG and total GWG among pregnant patients with overweight or obesity.

**Trial Registration:**

ClinicalTrials.gov Identifier: NCT03880461

## Key Points

**Question:**

Does standard care plus an evidence-based, adaptive mobile health lifestyle intervention reduce excessive gestational weight gain (GWG) for patients with overweight or obesity compared with standard care alone?

**Findings:**

In this cluster-randomized trial including 58 randomized clinicians treating 1265 enrolled pregnant patients, compared with standard care, the intervention significantly lowered the weekly rate of GWG and total GWG. Fewer patients in the intervention group than in the standard care group exceeded the national guidelines for weekly rate of GWG and for total GWG.

**Meaning:**

This cluster-randomized clinical trial demonstrated that an evidence-based, mobile health lifestyle intervention modestly reduced total GWG and weekly rate of GWG among patients with overweight or obesity.

## Teaser

This cluster-randomized clinical trial tests the effect of an adaptive mobile health lifestyle intervention plus standard care on gestational weight gain vs standard care alone among pregnant patients with overweight or obesity.

## Introduction

More than 60% of pregnant individuals in the US enter pregnancy with overweight or obesity.^1^ Of those individuals, more than half exceed the gestational weight gain (GWG) guidelines based on body mass index (BMI; calculated as weight in kilograms divided by height in meters squared) of the Institute of Medicine (IOM, now the National Academy of Medicine)^1,2^; this further increases their elevated risk of perinatal complications.^3,4^ The US Preventive Services Task Force (USPSTF) found intensive behavioral counseling interventions effectively reduced GWG^5^ and recommended that clinicians offer them.^6^ However, delivering behavioral interventions is resource intensive and impractical in routine care,^5^ emphasizing the need for scalable strategies to prevent excess GWG in patients with overweight or obesity.^6^

Compared with resource-intensive programs requiring multiple in-person sessions, mobile health (mHealth) interventions may be more accessible and cost-effective,^7^ potentially improving uptake and scalability. Adaptive (or stepped-care) interventions, which adjust intensity based on patient response,^8,9^ conserve resources for patients with the greatest need, increasing scalability. To our knowledge, prior trials have not tested large-scale, adaptive mHealth interventions in clinical settings or integrated technology providing real-time, automated, and personalized feedback to patients and clinicians—features that may enhance clinical application, scalability, and translation.

The Lifestyle, Eating, Activity in Pregnancy (LEAP) cluster-randomized clinical trial, with randomization at the clinician level, tested a multilevel, adaptive mHealth intervention plus standard care for pregnant patients with overweight or obesity. Primary outcomes were weekly rate of and total GWG, both continuous and categorized per IOM guidelines.

## Methods

This cluster-randomized clinical trial was approved by the Kaiser Permanente Northern California (KPNC) institutional review board. All clinicians and patients provided informed consent electronically. The rationale and methods of the LEAP cluster randomized trial are described elsewhere,^10^ and the trial protocol and statistical analysis plan are presented in Supplement 1. This study is reported following the Consolidated Standards of Reporting Trials (CONSORT) reporting guideline.

### Setting and Participants

The trial was conducted at 4 medical centers (Oakland, San Francisco, Redwood City, and Santa Clara) of KPNC, an integrated health system. Obstetric clinicians were invited to participate if they had a panel size of at least 5 patients with overweight or obesity and planned to stay at KPNC during the trial period. Interested clinicians completed a brief online survey and consented between October and December 2020 (Figure). Pregnant patients of enrolled clinicians were eligible to be recruited between January 2021 and October 2023 if they were at 8 to 15 gestational weeks, aged at least 21 years, English-speaking, had a singleton pregnancy, had a BMI of 25 to <40 (patients with BMI ≥40 were excluded due to their extremely high obstetric risk), had smartphone and Wi-Fi access, and planned to deliver at KPNC.

**Figure.**
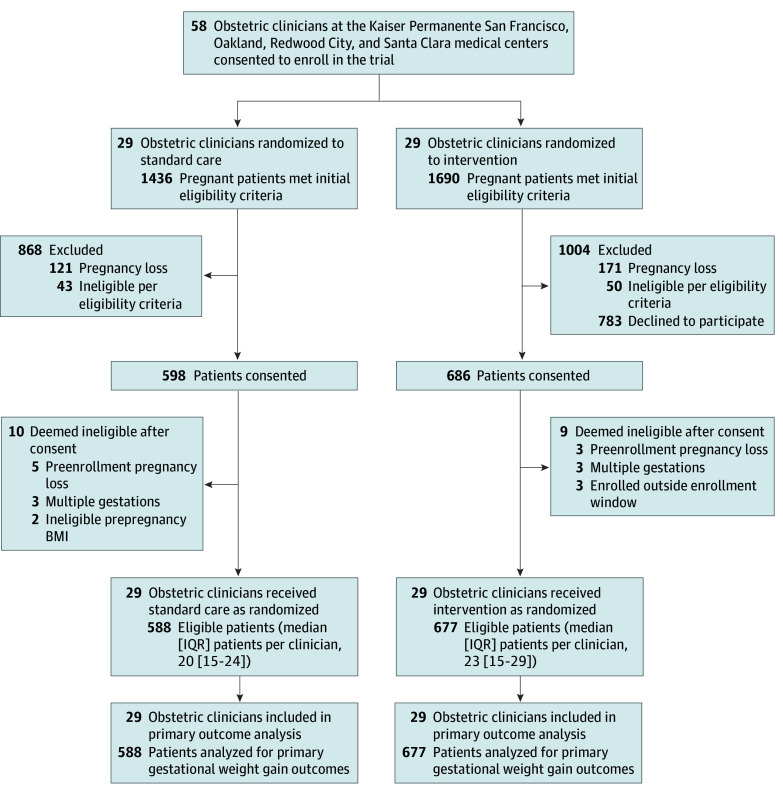
LEAP Cluster-Randomized Clinical Trial Flowchart BMI indicates body mass index.

### Randomization and Blinding

Cluster randomization was performed at the clinician level, with each cluster defined as a participating clinician and their eligible patients. Clinicians were randomized 1:1 to standard care or intervention conditions. A covariate-constrained randomization scheme was implemented to reduce the chance of between-condition imbalance on important covariates, including patient-level factors, by eliminating all allocations to the intervention and standard care conditions that did not meet specified balance criteria from among all possible allocations.^10,11^ Covariates for balance included clinician age, patient self-reported race and ethnicity (Asian, Black, Hispanic, White, and other [eg, American Indian or Alaska Native, Native Hawaiian or Other Pacific Islander, multiple races, and unknown race or ethnicity]), patient BMI (overweight or obesity), and number of eligible patients in their panel. Race and ethnicity were treated as social constructs shaped by historical and contemporary racism in the US.^12^

According to the cluster-randomized design, patients’ randomized condition corresponded to that of their clinician. The biostatistician, study investigators, and survey reminder staff were blinded to condition.

#### Standard Care

Clinicians in the standard care group received quarterly newsletters on trial recruitment and retention. Per routine care, clinicians generate patients’ GWG graphs in relation to the IOM guidelines using weights in the electronic health record (EHR) at the 20-week prenatal visit. Patients received standard prenatal care (approximately 8 prenatal visits). Medical staff routinely weigh patients at each visit and provide a newsletter describing the IOM guidelines and healthy eating tips at the first visit.

#### Intervention

Clinicians in the intervention group received the same quarterly trial newsletters plus additional newsletters on motivational interviewing for discussing GWG. They were encouraged to use the EHR-generated GWG graph to discuss GWG in each trimester.

Patients of intervention condition clinicians were offered the LEAP program, a structured mHealth lifestyle intervention adapted from prior successful trials^13,14^ and described elsewhere.^10^ Interested patients consented to the program, which promoted meeting IOM GWG guidelines, healthy eating, gradually increasing activity to 150 minutes of moderate to intensity activity per week, and building skills to manage barriers to healthy behaviors. Patients received personalized, automated feedback on GWG and activity via a study smartphone app, linked to a cellular digital scale (model BT005 or BT006; BodyTrace) and activity tracker (Fitbit [Google] or Apple Watch [Apple]); 13 educational topics released weekly in the app; and step-wise support from a human lifestyle coach (a registered dietitian) via chat or telephone. Patients were encouraged to use the app and self-monitoring tools and received step-wise support from enrollment until delivery. Intervention components were designed to support self-monitoring, goal setting, and feedback on goal attainment.

LEAP followed an adaptive, stepped-care model, reserving higher-intensity interventions for patients experiencing accelerated GWG. All patients received step 1: a personalized calorie target developed in an initial video visit with the human coach, the 13 weekly education topics, and encouragement to track weight, activity, and diet from enrollment until delivery. Movement to higher steps was based on weight trajectory per the digital scale. For GWG between the 75th percentile and upper IOM limit, step 2 added weekly personalized chat messages from the coach. If GWG exceeded the upper IOM limit, step 3 added biweekly telephone sessions with the coach. Calorie goals in steps 2 and 3 were adjusted as needed.^10^

Regarding intervention fidelity, coaches received standardized training and supervision; all human-delivered intervention materials had corresponding fidelity checklists. Review of 10% of calls between coaches and patients found 90.0% fidelity for step 1 calls and 77.2% fidelity for step 3 calls. Five newsletters delivering intervention content were emailed to intervention clinicians; 97% enrolled clinicians were sent the first newsletter, and 86% were sent newsletters 2 through 5. Missed newsletters were due to 1 faulty email address and 3 clinician departures.

### Outcome Assessment

#### Primary Outcomes

All primary outcomes were obtained from the EHR. Prepregnancy weight was defined as a weight measured in pregnancy up to 10 gestational weeks (available for 94.6% of patients), or, if missing, the prepregnancy weight measured closest to pregnancy, within the previous 12 months (used for 5.4% of patients). Mean weekly rate of GWG (kilograms per week) was calculated as total GWG (last measured pregnancy weight minus prepregnancy weight) divided by the number of weeks between the date of the last menstrual period and last measured pregnancy weight. Weekly rate of GWG was categorized per the IOM BMI-specific guidelines^15^ for rate of GWG as exceeding, meeting, or below the recommended range.^10^ The IOM’s BMI-specific guidelines for weekly rate of GWG include a range for weight gain during the first trimester (0.5-2.0 kg, regardless of BMI) along with BMI-specific ranges for weight gain per week during the second and third trimesters (0.23-0.33 kg for overweight, 0.17-0. 27 kg for obesity). Exceeding the weekly rate of GWG according to IOM guidelines was defined as being above the sum of the upper limit for the first trimester (2.0 kg) plus the upper limit for the weekly rate (0.33 kg for overweight, 0.27 kg for obesity) multiplied by the number of weeks in the second and third trimesters (ie, until the last measured pregnancy weight). Being below the IOM guidelines for weekly rate of GWG was defined as being below the sum of the lower limit for the first trimester (0.5 kg) plus the lower limit for the weekly rate (0.23 kg for overweight, 0.17 kg for obesity) multiplied by the number of weeks in the second and third trimesters (ie, until the last measured pregnancy weight). Meeting the recommendations was defined as being within the IOM cut points. Data were included regardless of gestational age at delivery.

To assess GWG during the period of exposure to the intervention, we also calculated weekly rate of GWG (kilograms per week) as postenrollment weight gain (last measured weight minus first measured weight after enrollment) divided by the number of weeks between the date of these 2 weights. This weekly rate of GWG was also categorized according to the IOM guidelines.

Total GWG (kilograms) was calculated as a last measured weight (within 3 weeks of delivery) minus prepregnancy weight. Total GWG was categorized per the IOM guidelines for total GWG as exceeding, meeting, or below the recommended range. The IOM’s BMI-specific guidelines for total gestational weight gain provide recommended ranges of 7 to 11.5 kg for individuals with overweight and 5 to 9 kg for individuals with obesity. Exceeding the IOM guidelines for total GWG was defined as having a total gain above the upper limit of the IOM range (11.5 kg for overweight, 9 kg for obesity). Being below the IOM guidelines for total GWG was defined as having a total gain below the lower limit of the IOM range (7 kg for overweight, 5 kg for obesity). Meeting the recommendations was defined as being within the IOM cut points described above.

#### Secondary Outcomes

##### Infant Birth Weight

Outcomes were ascertained from the EHR and included mean birth weight in grams. Categorical outcomes captured both extremes of fetal growth, including large for gestational age (LGA; >90th and >95th percentiles) and small for gestational age (SGA; <10th and <5th percentiles), based on US sex- and gestational age–specific reference distributions,^16^ as well as macrosomia (>4000 g), and low birthweight (<2500 g).

##### Health Behaviors

Validated surveys were administered at baseline (10 to <15 weeks’ gestation) and follow-up (33-39 weeks’ gestation). Diet quality was measured with the Block Food Frequency Questionnaire and calculated using the Alternate Healthy Eating Index for Pregnancy.^17^ Physical activity was assessed with the Pregnancy Physical Activity Questionnaire. Frequency of self-weighing was assessed via self-report, with an item adapted from prior trials,^18^ and objectively with the study-provided digital scale for intervention patients.

#### Adverse Outcomes

Adverse outcomes included pregnancy loss; preterm delivery (<37 weeks); extreme (<28 weeks), very (28-32 weeks), and late (32 to <37 weeks) preterm delivery; primary cesarean section; gestational hypertension; preeclampsia or eclampsia; gestational diabetes; coronary arterial disease; neonatal intensive care unit admission; and perinatal depression, all obtained from the EHR. Self-reported fractures or sprains were assessed via survey.

### Statistical Analysis

All data analyses were intention-to-treat, including all randomized clinicians and their consented patients. Analysis of treatment condition differences were by original treatment assignment, regardless of participation and adherence rates to the intervention. Power and sample size are described elsewhere.^10^

We used log binomial regression to estimate the population mean intervention effect on binary outcomes, including exceeding the IOM guidelines (primary outcomes) and infant size for gestational age (secondary outcome). Parameter estimation was by generalized estimating equations (GEE; marginal model) accounting for the within-clinician correlation among patients to obtain valid estimates of relative risks (RRs) and associated 95% CIs. Similarly, we used linear regression with estimation via GEE to provide point and interval estimates of between-condition differences in means for continuous outcomes, including rate of GWG and total GWG (primary outcomes) and changes in diet quality, changes in physical activity, and birth weight (secondary outcomes). The a priori specified set of model covariates included those used in the randomization procedure (clinician age, patient race and ethnicity, and patient BMI) and patient age, given their strong associations with GWG. We used multiple imputation for handling missing data with the fully conditional specification method to generate 50 imputed datasets, and we used the predictive mean matching method to impute missing values for continuous variables.^19,20^ Regression analyses were performed on each imputed dataset, with results combined using Rubin rules^21^ providing valid point and interval estimates appropriately accounting for the uncertainty in imputing the missing data.

Two exploratory analyses assessed intervention effectiveness across levels of engagement with self-weighing. We used an instrumental variable to estimate the average causal effect among adherent patients, a measure of the effectiveness of the intervention among those who adhered to assigned treatment, with the randomization indicator used as the instrumental variable.^22^ For this analysis, all or none adherence was defined as self-monitoring weight more than 5 times per week, per the scale.^23^ A key applicable assumption was that outcomes among patients who were not adherent were not affected by randomized assignment.

*P* values were 2-sided, and statistical significance was set at *P* ≤ .05. Analyses were conducted using SAS software version 9.4 (SAS Institute) from May 1, 2024, to February 12, 2026.

## Results

The final analytic sample included 58 clinicians (29 per group) and 1265 patients (mean [SD] age, 33.4 (4.6) years; mean [SD] prepregnancy BMI, 29.8 [3.8]), with 588 patients in the standard care group and 677 patients in the intervention group. Self-reported race and ethnicity data revealed a diverse sample: 293 Asian patients (23.2%), 74 Black patients (5.9%), 331 Hispanic patients (26.2%), 430 White patients (34.0%), and 137 patients (10.8%) who identified as other race or ethnicity (Table 1). The Figure summarizes the flow of participants through the trial. Patient characteristics were similar across groups, except that more intervention patients were nulliparous (303 patients [44.8%] vs 223 patients [37.9%]). Patients who enrolled to the LEAP program were less likely to be non-Hispanic Black or Hispanic than those who did not enroll (eTable 1 in Supplement 2). The enrolled patients who agreed to participate in the LEAP intervention program were slightly more educated than enrolled patients who did not participate in the intervention (eTable 2 in Supplement 2).

**Table 1.** Clinician and Patient Baseline Characteristics by Treatment Condition

Characteristic	No. (%)		
	Standard care	Intervention	Overall
Clinician characteristics^a^			
No.	29	29	58
Medical center			
Oakland	9 (31.0)	9 (31.0)	18 (31.0)
Redwood City	5 (17.2)	6 (20.7)	11 (19.0)
Santa Clara	10 (34.5)	10 (34.5)	20 (34.5)
San Francisco	5 (17.2)	4 (13.8)	9 (15.5)
Age, mean (SD), y	47.7 (5.4)	46.8 (8.7)	47.2 (7.2)
Eligible patient panel size, mean (SD), No.	28.6 (12.4)	28.6 (15.1)	28.6 (13.7)
Patient characteristics			
No.	588	677	1265
Age at enrollment, y			
Mean (SD)	33.5 (4.5)	33.3 (4.7)	33.4 (4.6)
21-29	116 (19.7)	145 (21.4)	261 (20.6)
30-34	224 (38.1)	250 (36.9)	474 (37.5)
35-39	203 (34.5)	224 (33.1)	427 (33.8)
40-46	45 (7.7)	58 (8.6)	103 (8.1)
Race and ethnicity			
Asian	129 (21.9)	164 (24.2)	293 (23.2)
Black	37 (6.3)	37 (5.5)	74 (5.9)
Hispanic	154 (26.2)	177 (26.1)	331 (26.2)
White	201 (34.2)	229 (33.8)	430 (34.0)
Other^b^	67 (11.4)	70 (10.3)	137 (10.8)
Parity			
Nulliparous	223 (37.9)	303 (44.8)	526 (41.6)
Primiparous	228 (38.8)	253 (37.4)	481 (38.0)
Multiparous	120 (20.4)	99 (14.6)	219 (17.3)
Missing	17 (2.9)	22 (3.3)	39 (3.1)
Prepregnancy weight, mean (SD), kg^c^	79.3 (12.5)	78.2 (12.5)	78.7 (12.5)
Prepregnancy BMI			
Mean (SD)	29.9 (3.9)	29.7 (3.8)	29.8 (3.8)
25.0-29.9	338 (57.5)	410 (60.6)	748 (59.1)
30.0-39.9	250 (42.5)	267 (39.4)	517 (40.9)
Gestational age at enrollment, mean (SD), wk	10.2 (1.9)	10.3 (1.9)	10.3 (1.9)

Abbreviation: BMI, body mass index (calculated as weight in kilograms divided by height in meters squared).

^a^ Clinician characteristics were based on data in the 1 year prior to randomization.

^b^ Includes American Indian or Alaska Native, Native Hawaiian or Other Pacific Islander, multiple races, and unknown race or ethnicity.

^c^ Prepregnancy weight was defined as either the first measured weight during pregnancy up to 10 weeks’ gestation (632 intervention patients [93.4%]; 563 standard care patients [95.8%]), or the last measured weight within the 12 months prior to pregnancy (45 intervention patients [6.7%]; 25 standard care patients [4.3%]).

### Primary Outcomes

Patients of clinicians in the intervention group had a lower mean weekly rate of GWG than those in standard care group (mean [SD] rate, 0.25 [0.20] kg/week vs 0.28 [0.20] kg/week; mean between-group difference, −0.03 [95% CI, −0.05 to −0.01] kg/week) (Table 2). The proportion exceeding IOM guidelines for weekly rate of GWG was lower in the intervention group vs standard care group (351 patients [51.9%] vs 354 patients [60.2%]; RR, 0.86 [95% CI, 0.78 to 0.95]); whereas the proportion below the guidelines for weekly rate of GWG was higher in the intervention group than the standard care group (148 patients [21.8%] vs 101 patients [17.2%]; RR, 1.27 [95% CI, 1.01 to 1.61]) (Table 2). In postenrollment analyses restricted to the period of intervention exposure, the intervention group was significantly more likely to gain below IOM-recommended ranges, whereas exceeding IOM-recommended ranges did not differ significantly between groups (RR, 0.92 [95% CI, 0.85 to 1.00]) (Table 2). Patients of intervention clinicians had lower total GWG than those of standard care clinicians (mean [SD] change, 9.7 [6.2] kg vs 10.6 [6.2] kg; mean between-group difference, −0.87 [95% CI, −1.40 to −0.34]) (Table 2). The proportion of patients exceeding IOM guidelines for total GWG was lower in the intervention group vs standard care group (299 patients [44.1%] vs 301 patients [51.2%]; RR, 0.87 [95% CI, 0.77 to 0.98]), and intervention patients were more likely than standard care patients to gain below the IOM guidelines (192 patients [26.8%] vs 126 patients [21.5%]; RR, 1.23 [95% CI, 1.04 to 1.45]) (Table 2).

**Table 2.** Primary GWG Outcomes by Treatment Condition

Outcome	Standard care	Intervention	Between-condition difference in means (95% CI)^a^	Relative risk (95% CI)^a^
**Among all patients**				
No.	588	677	NA	NA
Entire pregnancy				
Weekly rate of GWG, mean (SD), kg/wk	0.28 (0.20)	0.25 (0.20)	−0.03 (−0.05 to −0.01)	NA
Compared with IOM-recommended weekly GWG rate, No. (%)				
Exceeding	354 (60.2)	351 (51.9)	NA	0.86 (0.78 to 0.95)
Meeting	133 (22.5)	178 (26.3)	NA	1.16 (0.96 to 1.40)
Below	101 (17.2)	148 (21.8)	NA	1.27 (1.01 to 1.61)
Total GWG, mean (SD), kg	10.6 (6.2)	9.7 (6.2)	−0.87 (−1.40 to −0.34)	NA
Compared with IOM-recommended total GWG, No. (%)				
Exceeding	301 (51.2)	299 (44.1)	NA	0.87 (0.77 to 0.98)
Meeting	161 (27.4)	197 (29.1)	NA	1.07 (0.86 to 1.32)
Below	126 (21.5)	182 (26.8)	NA	1.23 (1.04 to 1.45)
During second and third trimesters				
Weekly rate of GWG, mean (SD), kg/wk	0.40 (0.53)	0.36 (0.59)	−0.05 (−0.12 to 0.03)	NA
Compared with IOM-recommended weekly GWG rate, No. (%)				
Exceeding	430 (73.0)	464 (68.6)	NA	0.92 (0.85 to 1.00)
Meeting	81 (13.8)	91 (13.4)	NA	0.99 (0.75 to 1.30)
Below	77 (13.1)	122 (18.1)	NA	1.40 (1.06 to 1.85)
**Among patients with BMI 25.0 to 29.9**				
No.	338	410	NA	NA
Entire pregnancy				
Weekly rate of GWG, mean (SD), kg/wk	0.31 (0.19)	0.27 (0.20)	−0.03 (−0.07 to 0.00)	NA
Compared with IOM-recommended weekly GWG rate, No. (%)				
Exceeding	221 (65.5)	221 (54.0)	NA	0.84 (0.75 to 0.95)
Meeting	74 (21.9)	108 (26.4)	NA	1.23 (0.97 to 1.58)
Below	43 (12.6)	81 (19.7)	NA	1.51 (1.09 to 2.09)
Total GWG, kg	11.8 (5.8)	10.6 (6.1)	−0.98 (−1.69 to −0.26)	NA
Compared with IOM-recommended total GWG, No. (%)				
Exceeding	180 (53.3)	180 (43.9)	NA	0.83 (0.71 to 0.97)
Meeting	101 (29.9)	126 (30.7)	NA	1.03 (0.79 to 1.32)
Below	57 (16.8)	104 (25.3)	NA	1.42 (1.13 to 1.79)
During second and third trimester				
Weekly rate of GWG, mean (SD), kg/wk	0.44 (0.48)	0.39 (0.57)	−0.04 (−0.14 to 0.05)	NA
Compared with IOM-recommended weekly GWG rate, No. (%)				
Exceeding	253 (74.8)	285 (69.4)	NA	0.93 (0.85 to 1.02)
Meeting	47 (13.8)	55 (13.3)	NA	0.98 (0.67 to 1.43)
Below	39 (11.4)	71 (17.3)	NA	1.51 (1.04 to 2.19)
**Among patients with BMI 30.0 to 39.9**				
No.	250	267	NA	NA
Entire pregnancy				
Weekly rate of GWG, mean (SD), kg/wk	0.23 (0.19)	0.21 (0.19)	−0.02 (−0.05 to 0.02)	NA
Compared with IOM-recommended weekly GWG rate, No. (%)				
Exceeding	133 (53.1)	130 (48.7)	NA	0.93 (0.79 to 1.10)
Meeting	59 (23.4)	70 (26.2)	NA	1.06 (0.78 to 1.42)
Below	59 (23.5)	67 (25.0)	NA	1.06 (0.77 to 1.46)
Total GWG, mean (SD), kg	9.1 (6.4)	8.5 (6.2)	−0.51 (−1.42 to 0.40)	NA
Compared with IOM-recommended total GWG, No. (%)				
Exceeding	121 (48.3)	119 (44.4)	NA	0.95 (0.79 to 1.15)
Meeting	60 (24.0)	71 (26.5)	NA	1.06 (0.75 to 1.51)
Below	69 (27.7)	78 (29.1)	NA	1.03 (0.81 to 1.31)
During second and third trimester				
Weekly rate of GWG, mean (SD), kg/wk	0.34 (0.58)	0.30 (0.60)	−0.04 (−0.18 to 0.09)	NA
Compared with IOM-recommended weekly GWG rate, No. (%)				
Exceeding	174 (69.7)	176 (66.0)	NA	0.93 (0.82 to 1.05)
Meeting	34 (13.7)	35 (13.1)	NA	0.97 (0.63 to 1.47)
Below	42 (16.6)	56 (20.9)	NA	1.25 (0.84 to 1.86)

Abbreviations: GWG, gestational weight gain; IOM, Institute of Medicine; NA, not applicable.

^a^ All models were specified using the standard care group as the referent and were adjusted for prepregnancy body mass index, race and ethnicity, clinician facility, clinician age, maternal age, prepregnancy weight, and parity. Models for total GWG were additionally adjusted for gestational age at delivery.

There were no significant interactions by prepregnancy BMI, race and ethnicity, or neighborhood deprivation index. Stratified analyses showed larger between-group differences in total GWG in patients with overweight but not among patients with obesity (Table 2).

### Secondary Outcomes and Adverse Outcomes

Infants in the intervention group had lower risk of being LGA (73 infants [11.7%] vs 86 infants [15.7%]; RR, 0.75 [95% CI, 0.58 to 0.98]) and no significantly increased risk of being SGA compared with the standard care group. Infant birth weight was significantly lower in the intervention group than the standard care group (mean [SD] birth weight, 3313.9 [610.4] vs 3380.5 [563.7] g; mean between-group difference, −60.4 [−105.8 to −15.1] g) (Table 3). There were no significant group differences in change in diet (eTable 3 in Supplement 2) or physical activity (eTable 4 in Supplement 2). Daily reported self-weighing increased from 7.9% at baseline to 29.6% at follow-up in intervention patients, while decreasing in standard care patients (11.1% to 7.6%) (eFigure 1 in Supplement 2). Assessed adverse outcomes did not differ by group (Table 4).

**Table 3.** Birth Weight Outcomes by Treatment Condition^a^

	Infants, No. (%)		Between-condition difference in means (95% CI)^b^	Relative risk (95% CI)^b^
	Standard care (n = 546)	Intervention (n = 621)		
Birthweight, mean (SD), g	3380.5 (563.7)	3313.9 (610.4)	−60.4 (−105.8 to −15.1)	NA
LGA, percentile				
>90th	86 (15.7)	73 (11.7)	NA	0.75 (0.58 to 0.98)
>95th	48 (8.9)	37 (5.9)	NA	0.68 (0.48 to 0.97)
SGA				
<10th	40 (7.4)	60 (9.6)	NA	1.22 (0.88 to 1.70)
<5th	22 (4.1)	30 (4.8)	NA	1.12 (0.73 to 1.72)
Fetal macrosomia (>4000 g)	67 (12.2)	64 (10.3)	NA	0.87 (0.65 to 1.16)
Low birth weight (<2500 g)	32 (5.8)	42 (6.8)	NA	1.28 (0.80 to 2.04)

Abbreviations: LGA, large for gestational age; NA, not applicable; SGA, small for gestational age.

^a^ Analyses included all eligible consented participants who delivered a live birth.

^b^ All models were specified using the standard care group as the referent and were adjusted for prepregnancy body mass index, race and ethnicity, clinician facility, clinician age, maternal age, and gestational age at delivery.

**Table 4.** Adverse Prenatal and Neonatal Outcomes by Treatment Condition

Outcome	Patients, No. (%)		Relative risk (95% CI)^a^
	Standard care (n = 588)	Intervention (n = 677)	
Adverse gestational outcomes			
Pregnancy loss	36 (6.1)	49 (7.2)	1.16 (0.83-1.63)
Still birth	5 (0.9)	5 (0.8)	0.88 (0.28-2.82)
Gestational diabetes^b^	65 (12.1)	62 (10.1)	0.80 (0.53-1.22)
Hypertensive disorders^c^^,^^d^			
Gestational hypertension	79 (14.3)	85 (13.4)	0.86 (0.64-1.17)
Preeclampsia or eclampsia	87 (15.7)	94 (14.9)	0.89 (0.69-1.16)
Coronary arterial disease^d^	2 (0.4)	1 (0.2)	0.60 (0.04-8.62)
Pregnancy depression^e^	50 (8.4)	49 (7.2)	0.86 (0.57-1.30)
Preterm delivery^f^			
Preterm (<37 wk)	44 (8.1)	50 (8.1)	0.96 (0.68-1.34)
Extreme preterm (<28 wk)	2 (0.4)	1 (0.2)	0.44 (0.04-4.41)
Very preterm (28 to <32 wk)	1 (0.2)	8 (1.3)	6.85 (0.93-50.6)
Late preterm (32 to <37 wk)	41 (7.5)	41 (6.6)	0.88 (0.59-1.31)
Cesarean section^f^	167 (30.5)	196 (31.4)	1.00 (0.87-1.15)
Primary cesarean section^g^	95 (21.2)	127 (24.3)	1.04 (0.84-1.28)
NICU admission^f^	44 (8.0)	48 (7.7)	0.88 (0.59-1.30)
Sprain during or following exercise	7 (1.5)	7 (1.6)	1.02 (0.38-2.72)
Fracture during or following exercise	0	0	NA

Abbreviations: NA, not applicable; NICU, neonatal intensive care unit.

^a^ All models were specified using the standard care group as the reference and were adjusted for prepregnancy body mass index, race and ethnicity, clinician facility, clinician age, maternal age, prepregnancy weight, and parity. Due to low event rates and to ensure model convergence, models for still birth, coronary arterial disease, extreme preterm, very preterm, sprain and fractures were unadjusted.

^b^ Included participants without preexisting type 2 diabetes and gestational age at least 24 weeks (533 standard care patients; 606 intervention patients). Gestational diabetes was defined according to criteria used in the Kaiser Permanente Northern California clinical setting as 2 or more abnormal values during a 100-g, 3-hour oral glucose tolerance test according to the Carpenter and Coustan criteria or fasting glucose value at least 95 mg/dL after a 50-g, 1-hour oral glucose challenge test at least 180 mg/dL.

^c^ Included participants with gestational age at least 20 weeks (n = 551 standard care patients; 628 intervention patients).

^d^ Hypertensive disorders and coronary arterial disease were defined based on *International Statistical Classification of Diseases and Related Health Problems, Tenth Revision* (*ICD-10*) codes.

^e^ Defined as Patient Health Questionnaire-9 score of 10 or greater during pregnancy.

^f^ Included patients with livebirths (546 standard care patients; 621 intervention patients).

^g^ Included patients with livebirths and without prior cesarean section (432 standard care patients; 499 intervention patients).

### Treatment Adherence and Fidelity

Among 677 identified patients in the intervention group, 655 patients remained eligible and were invited to participate; Among these, 383 patients (58.5%) consented and 350 patients (53.4%) downloaded the app. Among patients who downloaded the app, 285 (81.4%) completed at least 1 education session (mean [SD], 7.4 [4.7] sessions), including 84 (29.5%) who completed 1 to 3 sessions, 53 (18.6%) who completed 4 to 6 sessions, and 148 (51.9%) who completed 7 to 13 sessions. For self-monitoring, 331 patients (94.6%) tracked diet (189 patients [54.0%]), activity (303 patients [86.6%]), or weight weekly (309 patients [88.3%]). Mean (SD) intervention exposure duration was 25.4 (5.6) weeks. By the end of pregnancy, 171 patients (48.7%) remained in step 1, 33 patients (9.5%) progressed to step 2, and 146 patients (41.8%) ended in step 3.

For our instrumental variable analysis, high intervention adherence (weight monitoring >5 times/week) occurred in 127 patients (36.4%). There were no differences by race and ethnicity in high adherence, completion of at least 1 education session, or any self-monitoring at least once weekly.

### Exploratory Analyses

Instrumental variable estimates of the absolute group differences in rate of GWG among patients with high intervention adherence (defined as self-weighing >5 times/week, per scale data) vs standard care were almost triple the differences observed in the primary analysis: −0.08 kg/week vs −0.03 kg/week for rate of GWG. Similarly, patients in the intervention group with high adherence, compared with the standard care group, had decreased rates of participants exceeding the IOM guidelines for rate of GWG (−22.9% vs −8.3%), greater proportion of patients meeting the guidelines (10.5% vs 3.8%), and greater proportion of patients gaining below the guidelines (12.7% vs 4.6%). Similar trends were observed for total GWG.

In additional exploratory analyses, patients with high intervention adherence were significantly less likely to exceed the IOM guidelines than patients in standard care for both rate of GWG (RR, 0.79 [95% CI, 0.67 to 0.94]) and total GWG (RR, 0.78 [95% CI, 0.65 to 0.94]) (eFigure 2 in Supplement 3). Patients with high adherence, compared with the standard care group, had a lower rate of GWG (mean [SD] change, 0.24 [0.13] kg/week vs 0.28 [0.20] kg/week; adjusted mean between-condition difference, −0.04 [95% CI, −0.07 to −0.02] kg/week) and less total GWG (mean [SD] change, 9.3 [5.0] kg vs 10.6 [6.2] kg; adjusted mean between-condition difference −1.52 [95% CI, −2.37 to −0.68] kg).

## Discussion

In this cluster-randomized clinical trial of an adaptive mHealth lifestyle intervention, including motivational interviewing training for clinicians and a behavioral intervention for their patients with overweight or obesity, we found that the intervention was modestly effective at reducing GWG: it reduced mean weekly rate of GWG and total GWG, as well as the proportion of patients exceeding the IOM guidelines for rate of GWG and total GWG. The LEAP intervention leveraged technology to provide real-time feedback on GWG to patients and only provided a more intensive intervention to those experiencing the most accelerated GWG. Exploratory analysis showed a dose-response effect, with intervention patients most adherent to self-weighing estimated to have the largest improvements in GWG. This trial addressed the USPSTF recommendations to test practical, evidence-based lifestyle interventions in clinical practice to promote healthy GWG.^6^

Intervention effects on weekly and total GWG in this cluster-randomized trial were modest (mean differences of −0.03 kg/week and −0.87 kg, respectively) compared with individually randomized trials, such as GLOW (−0.07 [95% CI, −0.09 to −0.04] kg/week and −2.19 [95% CI, −3.26 to −1.12] kg, respectively)^14^ and LIFE-MOMS (mean difference in total GWG, −1.59 [95% CI: −2.18 to −0.99] kg),^24^ which enrolled motivated volunteers. LEAP was implemented in routine clinical practice, with minimal exclusions and clinician-level randomization; only 59% of eligible patients consented to the intervention, and effect estimates reflect outcomes among all patients of clinicians assigned to the intervention, regardless of whether patients participated in or adhered to the intervention. Consequently, observed intention-to-treat effects reflect dilution from incomplete uptake and may estimate more closely the effects of implementation into routine practice.

From a population health perspective, even small improvements in GWG among patients at high risk of adverse outcomes related to excess GWG may yield meaningful benefits at scale, given the high prevalence of excess GWG and downstream maternal and infant consequences. Instrumental variable analyses suggested effects would be at least 2-fold greater with engagement in self-weighing more than 5 times/week (mean difference in total GWG, −1.52 [95% CI, −2.37 to −0.68] kg), underscoring potential for substantially greater impact with improved engagement. LEAP’s effect exceeded that reported in a review of digital interventions among individuals with overweight or obesity (mean GWG reduction, 0.63 kg).^7^

The intervention’s effects on measured health behaviors were mixed. We observed no effect on diet or physical activity. This is consistent with a 2025 meta-analysis that found mHealth apps were associated with effective weight control among pregnant women with overweight or obesity but did not were not associated with physical activity.^25^ In LEAP, the intervention did significantly increase patients’ daily self-weighing, a key component. A systematic review of adults with overweight or obesity^26^ found self-monitoring via digital health was consistently associated with weight loss in behavioral obesity treatment. Indeed, exploratory analyses found the magnitude of the LEAP intervention’s effects increased with increasing adherence to self-weighing in a dose-response manner. Our findings suggest a potential greater impact if interventions improve patient motivation for self-weighing.

### Strengths and Limitations

Strengths of this trial included the cluster-randomized design, which enabled a multilevel intervention for both clinicians and patients. The trial also had very few exclusions, unlike many randomized trials that enroll highly selected participants. LEAP’s adaptive intervention allowed enrollment of a large number of patients by reserving human counseling for fewer participants. Additional strengths include the highly diverse patient population and the rigor of intention-to-treat analyses and blinding. We obtained all GWG and other primary outcome data from the EHR, bolstering the generalizability of the findings to clinical practice.

This study has some limitations. First, weight data obtained from the EHR may not have been as accurate as research-grade weight measurements, although this strengthened the trial’s pragmatic approach and relevance to clinical settings. Second, we relied on self-reported assessments of physical activity and diet, possibly influenced by social desirability bias and subject to measurement error, which may have hindered our ability to detect differences in these behaviors. Third, enrolled patients were less likely to be Hispanic than nonenrolled patients. Similarly, and consistent with other mHealth trials, enrolled patients participating in the intervention program were more educated than enrolled patients not participating. Efforts to increase engagement and acceptability in these subgroups are needed. Fourth, resource constraints prevented translating the app to languages other than English, and participants were required to have smartphones and Wi-Fi access (however, these criteria excluded <5% of eligible patients). While the app was developed after usability testing among patients from diverse backgrounds, effectiveness in non–English speakers and less educated patients remains uncertain.

## Conclusions

In this cluster-randomized clinical trial, the adaptive, mHealth LEAP intervention effectively reduced GWG among pregnant patients with overweight or obesity, while conserving resources for those most in need. This trial demonstrated the impact of a practical, evidence-based lifestyle intervention delivered in clinical practice to promote healthy GWG.

## Notes

Supplement 1.
Trial Protocol and Statistical Analysis Plan

Supplement 2. **eTable 1.** Characteristics of patients who enrolled vs. did not enroll patients in the LEAP program **eTable 2.** Characteristics of enrolled trial patients who agreed versus did not agree to participate in the LEAP intervention **eTable 3.** Mean change in diet from baseline to follow up with mean differences by treatment condition **eTable 4.** Mean change in physical activity from baseline to follow up with mean differences by treatment condition **eFigure 1.** Patient reported self-weighing from baseline to follow up, by treatment condition

Supplement 3. **eFigure 2.** Adjusted mean condition differences and relative risk estimating condition differences in meeting the IOM guidelines for GWG by number of self-weighing per week

Supplement 4.
Data Sharing Statement
