# Supplementary material for: Adaptive Mobile Health Intervention to Reduce Excess Gestational Weight Gain: A Cluster-Randomized Clinical Trial
Source: JAMA Netw Open. 2026 Apr 20;9(4):e268007. doi: 10.1001/jamanetworkopen.2026.8007 (PMC13096985; doi:10.1001/jamanetworkopen.2026.8007)
Supplement: Supplement 1. — Trial Protocol and Statistical Analysis Plan [file jamanetwopen-e268007-s001.pdf]

LEAP Study
PI: Monique Hedderson, Ph.D.
Co-Is: Assiamira Ferrara, M.D., Ph.D., Susan Brown, Ph.D.
Biostatistician: Charles P. Quesenberry, Ph.D.
Funded by National Institutes of Health

LEAP Study Protocol & Analysis Plan

SECTION 1……………………………………………………………………………page 2
a. **Final protocol**…………………………………………………………………..page 3
b. **Summary of changes**…………………………………………………………page 17

SECTION 2……………………………………………………………………………page 20
c. **Final statistical analysis plan**………………………………………………page 21
d. **Revised power and sample size requested by DSMB**…………………page 21

REFERENCES…………………………………………………………………………..page 22

SECTION 1

FINAL PROTOCOL
LEAP STUDY

INTRODUCTION

Sixty percent of pregnant patients in the U.S. are overweight or obese. Over half of pregnant patients with overweight or obesity exceed the Institute of Medicine (IOM) guidelines for gestational weight gain (GWG),[1,2] which further increases their elevated risk of gestational diabetes (GDM), cesarean delivery, postpartum weight retention, severe obesity later in life, having a large-for-gestational-age infant[3,4] and a child at risk of overweight/obesity.[5-7] Thus, improving GWG among patients with overweight or obesity is a public health priority.

The main goal of this study is to test the comparative effectiveness of a mobile health (mHealth) intervention to manage gestational weight gain (as recommended by the IOM.[8]) in pregnant patients with overweight or obesity in Kaiser Permanente Northern California. To achieve this goal, we will conduct a cluster randomized controlled trial at 4 medical centers (Oakland, Redwood City, San Francisco, and Santa Clara) within Kaiser Permanente's Northern California region. The trial will include 56 consented clinicians randomized to standard care or standard care plus an mHealth intervention. We anticipate these clinicians will provide care to 2,040 eligible pregnant patients (1,020 in each arm) with a pre-pregnancy BMI between 25.0 kg/m^2 and 40.0 kg/m^2, who will consent to participate. The sample will be selected based on the patients' first body weight in pregnancy (up to 10 gestational weeks), measured by a KPNC provider, or if unavailable, then the weight measured closest to pregnancy within the prior 12 months. Patients will be enrolled by 15 weeks of pregnancy. We propose an adaptive intervention that begins with an effective, yet low resource-intensive treatment and then provides incremental support and resources only to patients who need it. The intervention consists of pregnancy lifestyle curriculum that is delivered primarily via an mHealth application; biweekly chat messages and telephone check-ins with study dietitians trained in motivational interviewing techniques will be added on for patients in need of additional support. Patients assigned to standard care will receive standard prenatal medical care. The lifestyle intervention will be compared to standard medical care. Outcomes will be assessed through electronic medical record (EMR) data.

Primary Outcome Measures:

1. **Total GWG,** defined as a last measured weight (within 3 weeks of delivery) minus pre-pregnancy weight (measured in pregnancy up to 10 gestational weeks; if missing, then the weight measured closest to pregnancy within the year prior); restricted to 24 gestational weeks or later.

2. **Rate of GWG,** defined as [total GWG (kg) / weeks of gestation at delivery], with no restriction on gestational age

3. The **proportion of patients who exceed, meet, or are below the IOM GWG guidelines** for:
    a. the weekly rate of GWG
    b. total GWG

Secondary Outcome Measures:

1. **Birthweight (grams),** defined as (mean (SD) and centiles (large for gestational age (LGA) > 90th percentile, small for gestational age (SGA) < 10th, and LGA > 95th, SGA < 5th),[9] macrosomia (> 4,000 g) and low birthweight (< 2,500 g).

2. **Postpartum weight retention**, assessed at 6 weeks postpartum, will use weights measured between 4 and 6 weeks postpartum minus pre-pregnancy weight *because* this a time when 80% of patients return to their pre-pregnancy weight[10,1110,112,32,32,32,3] and it is less likely to be impacted by weight changes due to breastfeeding.

3. **Infant growth trajectory** (BMI z-score), assessed from birth to 12 months, will use weight and length obtained from the **EMR at birth**, 3-5 days, and 1, 2, 4, 6, and 12 months. Z-scores at birth and 6 and 12 months will be used in the models of infant growth.

4. **Trimester-specific weekly rate of GWG (kg/week)**, assessed from 0 - 13 gestational weeks; 14 - 26 gestational weeks; 27 - 40 gestational weeks.

5. **GWG trajectory throughout pregnancy** will use all measured weights in pregnancy and is assessed from 10 gestational weeks until delivery.

6. **Change in moderate to vigorous physical activity (MET hours/week)**, as measured by the Pregnancy Physical Activity Questionnaire (PPAQ).

7. **Overall diet quality** according to the Healthy Eating Index-2015 (HEI-2015)

SETTING

The setting is KPNC, a large group practice prepaid health plan of 54 Medical Centers with more than 4M members who are representative of the geographic area. Patients use the same medical center for general medicine, obstetrics and for their children's pediatric visits. This integrated system provides a unique opportunity for follow-up and retention. KPNC maintains complete clinical databases supplemented by clinical information recorded in the EMR. To meet the projected sample size, eligible clinicians and their patients will be selected from Kaiser Permanente Oakland, San Francisco, Redwood City, and Santa Clara facilities.

RECRUITMENT, ELIGIBILITY & EXCLUSION CRITERIA

Clinician Eligibility. Clinicians are included in the eligibility pool if they:

1. are practicing in the Department of Obstetrics and Gynecology at the Oakland, San Francisco, Redwood City, or Santa Clara Northern California Kaiser facilities;
2. are physicians (M.D. or D.O.) or nurse practitioners; and
3. have their own panel and had ≥ 5 eligible patients on their panel in 2019.

Clinician Exclusion Criteria. Clinicians who meet any of the following exclusion criteria during a review of the electronic medical records or during the online eligibility screening are excluded from participation.

1. Residents or other providers who do not plan to stay at Kaiser Permanente during the next three years (i.e., the study recruitment period)
2. Clinicians whose current status is 'inactive,' indicating that they were terminated, are retired, etc.
3. Clinicians with fewer than 5 pregnant patients with overweight/obesity in 2019.

Patient Eligibility. Pregnant patients are initially identified as soon as they book their first prenatal visit by searching the Kaiser Permanente electronic medical record system, and are included in the eligibility pool if they:

1. are less than 12 weeks at survey recruitment
2. are female
3. had a new pregnancy episode and were on a provider's panel at the onset of pregnancy
4. are receiving prenatal care at the Oakland, San Francisco, Redwood City, or Santa Clara Northern California Kaiser facilities
5. are ≥ 21 years of age
6. have a pre-pregnancy BMI between 25.0 and 39.9 kg/m^2 (in this context, pre-pregnancy weight is defined as the first measured weight after pregnancy, up to 10 gestational weeks; if that is not available, then the weight measured closest to pregnancy within the prior 12 months is used)
7. do not require an interpreter and are not on the No Contact List at the Division of Research
8. have a valid e-mail address and phone number in the EMR

We have an automated program that provides real time identification of first prenatal care visits, from which we extract current weight and last menstrual period from the EMR on the same day as the first prenatal visit. Pre-pregnancy BMI is identified by searching the EMR for height and weight measurements taken by a KPNC clinical staff.

Patient Exclusion Criteria. Patients who meet any of the following exclusion criteria during a review of their electronic medical records, during the online eligibility screening, or the recruitment screening call, are excluded from participation.

The following items are exclusion criteria because they are **medical conditions** that are associated with our ability to accurately measure outcome variables.

- Multiple gestation (pregnant with more than one fetus)
- Pregnancy loss (miscarriage, abortion)

The following are exclusion criteria because we need to retain enrolled participants in order for our results to be meaningful. These situations interfere with full participation in the study. For example, patients who live outside of the service area will not be delivering their babies at the Kaiser Permanente Northern California facility, which means we would not be able to obtain their outcome data from the EMR. Access to a smartphone is required as part of the mHealth intervention.

- Plans to move out of the geographic area before delivery
- No access to a smartphone and Wi-Fi

Additionally, patients will be excluded if they are non-English speaking.

Clinician Recruitment. To recruit clinicians, the principal investigator attends a virtual obstetric care clinician meeting at each medical center to explain the study and recruit clinicians. Clinicians are then asked to complete a brief online survey at enrollment. Completion of the baseline survey, which includes elements of informed consent such as the goals of the study and voluntary nature of participation, indicates consent to enroll in the trial. Clinicians will be asked to complete a second survey at the end of the study after the last enrolled patient has delivered.

The surveys inquire about strategies and resources that clinicians use to counsel their patients with overweight and obesity on GWG, and resources they would like to have. For clinicians randomized to the intervention group, Survey 2 also will include brief intervention evaluation questions.

Consented clinicians in both groups will also receive quarterly study update emails.

Additionally, intervention group clinicians periodically will receive email-tip sheets for addressing weight in pregnancy – including information on motivational interviewing. Prior to the 24-week prenatal appointment, enrolled intervention group patients will receive a text encouraging them to discuss their GWG with their clinician during their upcoming visit. Following the 24-week prenatal appt, a secure message will be sent through the EMR to encourage intervention group patients to weigh themselves regularly, engage in healthy lifestyle changes, and actively participate in the LEAP program.

RANDOMIZATION

Randomization will take place after clinician consent is obtained. A covariate constrained randomization scheme is implemented to reduce the chance of between-arm imbalance on important covariates, including patient-level factors, by eliminating all allocations to the intervention and standard care arms that do not meet specified balance criteria from among all possible treatment allocations of the 56 clinicians at 4 medical facilities.[12] Covariates for balance include clinician age, patient race/ethnicity (Asian, Black, Hispanic, White, Other), patient body mass index (BMI) (overweight, obese), and number of eligibles (to help ensure balance on trial enrollment), all based on data in the one year prior to randomization. These variables are chosen given the expected association with study outcomes and/or intervention engagement and level of between cluster variability which would increase the likelihood of between-arm imbalance.

Patient Recruitment (Survey). Eligible patients of consented clinicians in both groups are invited to complete two surveys: one at baseline (8 - 15 gestational weeks) and the second at ~33 gestational weeks. Patients are sent an initial recruitment email inviting them to join the study and one week later, the recruiter will send a follow-up recruitment letter and contact patients by telephone using an IRB-approved script. The call provides an opportunity for the patient to receive information about the study.

Patient Recruitment (Intervention).

After completing the first survey, intervention group patients are invited to consent online to the intervention. The intervention is an adaptive intervention. All consented patients receive Step 1: a Fitbit, wireless scale, and a smartphone app, as well as a one-time introduction session with a trained lifestyle coach to set their goals. Patients who are between the 75th and 100th percentile of the IOM GWG guidelines received weekly chat support from the coach (Step 2). Patients who are above the upper limit of the guidelines receive biweekly calls from the coach (Step 3) as well as biweekly chat support from Step 2.

Patients are sent an initial recruitment email inviting them to join the intervention lifestyle program and one week later, the recruiter will send a follow-up recruitment letter and contact patients by telephone using an IRB-approved script. The call provides an opportunity for the patient to receive information about the lifestyle program.

RETENTION

Strategies to maximize retention and follow up, and Incentives. We will maximize clinician retention by sending quarterly clinician newsletters. Clinicians do not receive an incentive for survey completion.

We will also offer up to $60 in Amazon gift cards to patients for completion of Survey 1 and up to $50 in Amazon gift cards for completion of Survey 2. Patients who leave KPNC can continue to participate in the study.

TRACKING SYSTEM

Contact information and key characteristics used for randomization will be entered into a secure ACCESS database. The database will document each patient's participation status, track data collection, and intervention contacts achieved and provide a means for the regular monitoring and reporting of trial progress.

STANDARD MEDICAL CARE

Patients receive standard KPNC prenatal medical care, which includes an initial prenatal visit at 7-10 weeks' gestation, as well as a newsletter containing the IOM GWG guidelines and advice on healthy eating. Patients with routine pregnancies then receive an additional seven prenatal visits between 16 weeks' gestation and delivery. Medical staff weigh patients at each visit per standard care.

LIFESTYLE INTERVENTION

In addition to standard care, consented intervention patients will receive a multi-component pregnancy lifestyle intervention. The intervention was adapted from the DPP[13] and our prior research[14,15] to be feasible among pregnant patients with overweight or obesity for possible adoption in a healthcare system setting. The intervention targets behavior changes for weight management, healthy eating, physical activity, and stress management to meet a trial goal of gaining within the IOM recommendations for gestational weight gain.

Introductory Technology Setup Call.

Upon consenting, patients are mailed a package containing the study devices (Fitbit physical activity tracker and BodyTrace wireless scale) and setup instructions. Patients complete a one-time, 45-minute video call with a trained staff member who explains the program goals, helps the patient set up the devices and mHealth application, and works together with the patient to

establish a personalized, achievable physical activity goal for the next week (such as 10 minutes of moderate activity per day, for seven days). During the week following this setup call, patients are asked to review the first weekly education topic in the mHealth application and complete the following daily tasks: weigh themselves, log all foods and caloric beverages to inform their calorie goal, and wear their activity tracker. Patients are asked not to make any changes to their normal dietary intake and activity to allow the lifestyle coach to evaluate the patient's baseline levels.

Lifestyle Coach Call and Initial Goal Setting

Each patient is assigned to one lifestyle coach. Approximately one week after the technology setup call, the lifestyle coach holds a 30–60-minute video call to review patients' motivations for joining the program and familiarity with the tools provided. The lifestyle coach then explores facilitators and barriers to engaging in lifestyle health behaviors and offers support for using the self-monitoring tools. The patient and coach review the baseline daily caloric intake, meal patterns, and physical activity together. An initial calorie goal is established collaboratively by assessing the baseline average calories consumed from meals and snacks for up to three days and evaluating these patterns in relation to the patient's current GWG weight trajectory. If the GWG weight trajectory is on track (i.e., when the patient is in the green zone as described above), this average is their calorie goal; if the trajectory is above the GWG rate recommendations (i.e., when the patient is in the yellow or pink zone as described above), the calorie goal is 200 calories less than their current average. Calorie goals are never set at less than 1,200 calories per day, even if the patient's average is less than that, for example, due to nausea.

The lifestyle coach and patient collaboratively review the patient's physical activity goal and adjust it in small increments, based on actual accrued physical activity minutes. As noted above, the overall goal is to achieve 150 minutes of moderate-to-vigorous-intensity activity per week during the program.

INTERVENTION CONTENT.

The LEAP trial is an adaptive, or stepped-care, intervention that promotes four goals: 1) meet IOM GWG recommendations, 2) eat a healthful diet in appropriate portion sizes, 3) gradually achieve 150 minutes per week of moderate- to vigorous-intensity physical activity, and 4) build skills to manage stress and other challenges that can get in the way of healthy lifestyle behaviors. All patients in the intervention arm receive a structured mHealth intervention with tools (Fitbit, wireless scale, and mHealth application) to facilitate self-monitoring, goal-setting, and automated feedback in relation to goal attainment.

The adaptive mHealth intervention is a phased approach to behavior change based on social cognitive theory (SCT)[16,17] and the Transtheoretical model (TTM).[18] In this adaptive approach,

all consented intervention patients receive "Step 1," This includes technology-based tools, i.e., a LEAP mHealth application linked to a physical activity tracker (a Fitbit, unless the patient prefers to use her own Apple Watch), food tracking application (the Fitbit or MyFitnessPal application), and a BodyTrace Wi-Fi smart scale. Step 1 also includes automated text messages and core lifestyle education topics released weekly in the application. Patients receive an introductory call for technology setup and program introduction from an unblinded intervention research assistant; one week later, patients receive a call from a trained lifestyle coach. During the latter call, the patient and coach review the daily caloric intake, meal patterns, and physical activity goal, and mutually set calorie and physical activity goals. The personalized calorie target considers factors such as the patient's previous average calorie intake and weight trajectory.

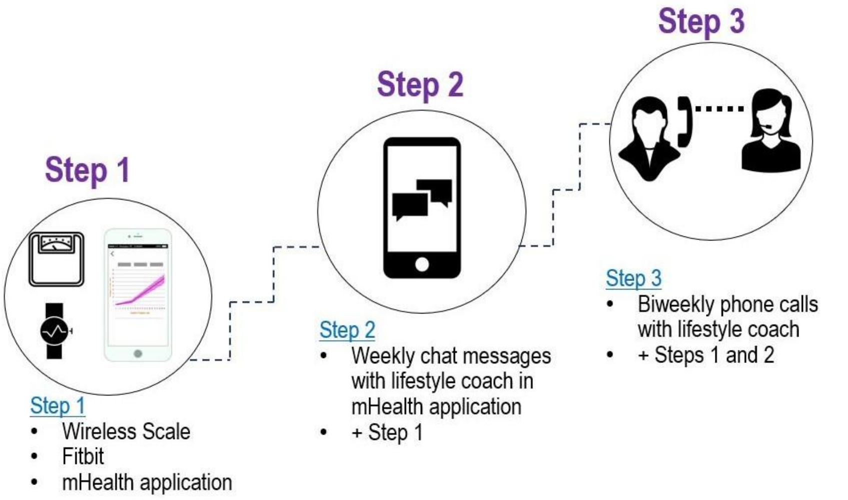

Step 1: Received by all patients; Step 2: Received by patients whose GWG is between the 75th percentile and upper limit of the IOM recommendations; Step 3: Received by patients whose GWG exceeds the upper limit of the IOM recommendations

Movement to a higher intervention Step is determined by observing ≥ three weights on separate days in a rolling 2-week period. For patients whose gestational weight gain is observed to be between the 75th percentile and upper limit of the IOM-recommended weight gain for their BMI, "Step 2" components are added; i.e., weekly chat messages from a lifestyle coach through the LEAP mHealth application. For patients whose weight gain reaches the upper limit of the IOM recommendations, "Step 3" components are further added; i.e., biweekly individual telephone sessions with a lifestyle coach. Calorie goals for patients at Step 2 and 3 are subsequently adjusted as needed by phone call or chat communication.

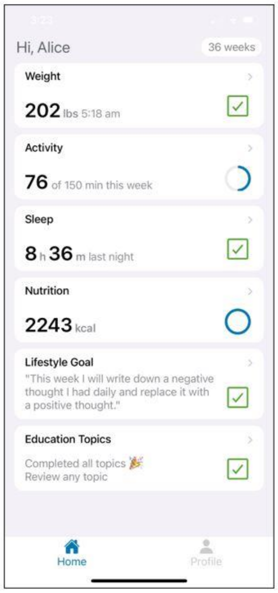

Education Topics and Lifestyle Goal Setting

The mHealth application includes education topics adapted from the Diabetes Prevention Program[19] and the prior GLOW randomized controlled trial targeting improved GWG among patients with overweight or obesity.[20] The education topics include the significance of healthy eating, including portion control, selecting nutrient-rich foods, and reducing added sugar and unhealthy fats. Patients also learn about the advantages of regular physical activity and ways to incorporate more movement into their daily routines, as well as stress reduction techniques and behavior change strategies like goal setting, self-monitoring, and problem-solving skills.

A new topic is released every week for the first 13 weeks of the program and prompts the patient to set a personally relevant, actionable lifestyle goal. Each topic provides three suggested options for weekly goals that are relevant to the topic. There is always an option for

patients to write in their own goal to tailor it to their needs. Weekly goals are then automatically populated from the education topic to the home page of the application as a visual reminder; patients are prompted to check off each goal once it is completed.

Intervention Step 1 Components

1. mHealth application

The mHealth smartphone application was created by Ejenta, Inc., a California-based technology company that develops systems for remote health care. All application content was developed by the LEAP trial team. The application is available to download for consented patients with an Apple iPhone or Android smartphone. The main screen of the application has different tiles (Weight, Activity, Sleep, Nutrition, Lifestyle Goal, Education Topics) that patients can click on for additional details, as described below.

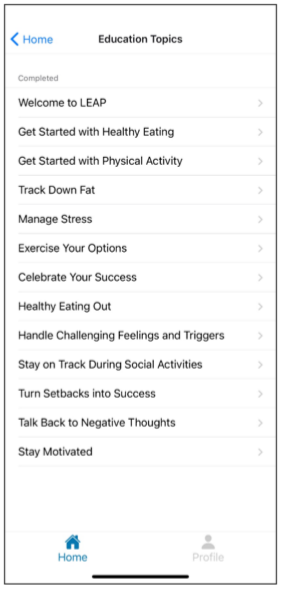

2. Weight Tracking

Patients are sent a BodyTrace wireless scale that transmits weights to the mHealth application in real-time via cellular technology. They are asked to weigh themselves at the same time daily using this scale. Weights are displayed in a graph that shows their current Step in the adaptive mhealth intervention: the green zone for Step 1, yellow for Step 2, and pink for Step 3. Patients are asked to review their weight gain graph daily. A minimum of three weights in a particular zone, collected on separate days across a rolling 2-week period, triggers movement to a higher Step. To eliminate erroneous data transmitted by the BodyTrace scale (e.g., other family members using the scale), implausible weights that are greater than or less than the following thresholds and collected across the specified time periods are automatically filtered out:

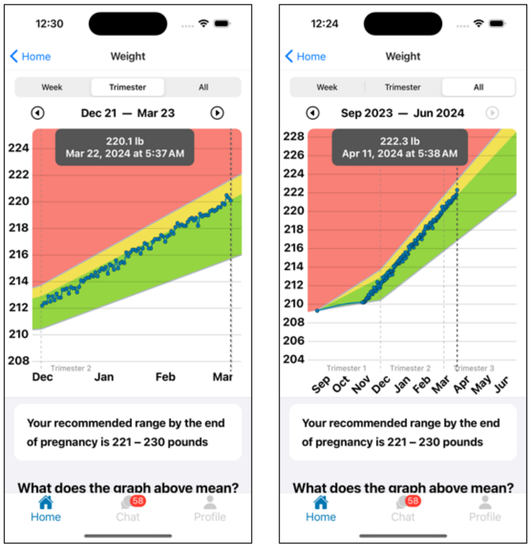

**Time period**	**Weight threshold** *Kilograms (Pounds)*
< 24 hours	5 (11)
24 - 48 hours	7.5 (16.5)
48 - 168 hours	10 (22)
168 - 336 hours	15 (33)
> 336 hours	20 (44)

3. Activity Tracking

Patients are mailed a Fitbit Charge 4 physical activity tracker and asked to wear it daily on their non-dominant wrist. Alternatively, patients who already own a Fitbit (any wrist-worn model) or an Apple Watch may choose to use their own device, which can be similarly integrated with the LEAP mHealth application.

Activity from the patient's tracker is transmitted in real-time to the mHealth application, where patients can view their progress toward their weekly physical activity goal. Patients are asked to select realistic goals to gradually work toward 150 minutes/week of moderate- to vigorous-intensity activity[21] during the program, in accordance with ACOG recommendations. Physical activity recommendations will be tailored to each woman's health status, preferences, and need to minimize injury and maximize the convenience, safety, and enjoyment of physical activity.

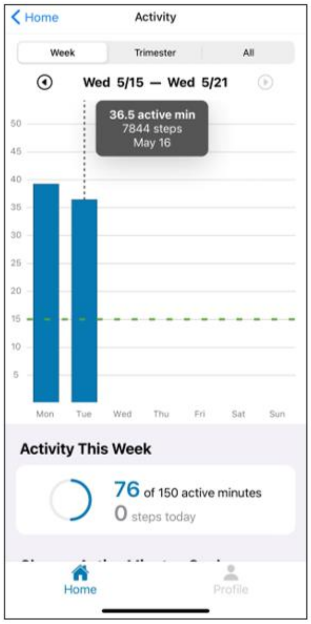

4. Sleep Tracking

Patients who wear their tracker while sleeping are able to view their sleep data in the mHealth application. The lifestyle coach discusses how sleep is related to other healthy behaviors; for example, how physical activity can improve sleep, or ways in which inadequate sleep may lead to less healthful eating choices.

5. Nutrition Tracking

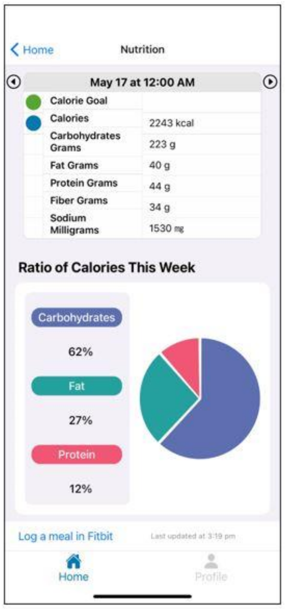

Patients are asked to track their food and beverage intake on a daily basis, or as often as possible, using the Fitbit app or the MyFitnessPal app; both transmit nutrition data to the LEAP mHealth application, and display the total daily calories consumed as well as weekly proportions of macronutrients. Patients are asked to review their nutrition data regularly so that they can compare their intake to their calorie goal and make adjustments to intake as needed to achieve their weight goals. Nutrition topics such as portion sizes, meal timing, and increasing fiber are discussed in the introduction call and weekly educational topics.

The emphasis will be on overall “healthy eating” versus a restrictive “diet,” i.e., eating more healthy vegetables, fruits, lean protein and high fiber foods while reducing fat and added sugars. The use of monounsaturated fats and complex carbohydrates will be recommended. Coaches will also promote awareness of portion sizes and choosing healthier options in support of lowering overall energy intake. The diet will be tailored to patients’ needs, access to healthy foods, and cultural preferences.

Intervention Step 2 Components

Patients whose GWG is between the 75th percentile and upper limit of the IOM GWG recommendations receive Step 2 of the LEAP intervention. In Step 2, patients receive weekly chat messages through the mHealth app from their lifestyle coach, in addition to Step 1 components. The lifestyle coach uses weight, diet, and activity data automatically transmitted to a research dashboard that derives its data from the mHealth application. Based on this information, the coach tailors messages to patients' behaviors and weight trajectory. Calorie goals are adjusted (+/- 200 kcals) as needed. These messages aim to enhance self-efficacy by acknowledging successful experiences in meeting short-term goals. Additionally, the messages provide tips for overcoming barriers and suggest small-step goals. Patients receive a notification when the lifestyle coach sends a message, and they have the option to respond to these messages within the chat if they have any questions or concerns. All messages remain in the chat so that patients can refer back to them.

Intervention Step 3 Components

Patients whose GWG reaches the upper limit of the IOM GWG recommendations receive Step 3 of the LEAP intervention. Patients receive biweekly 15-minute telephone coaching calls from their lifestyle coach, in addition to the Step 2 biweekly chat messages and Step 1 components. In Step 3 calls, short-term physical activity (PA) and dietary goals are reviewed based on the collected data and previous success in meeting goals. Weekly goals are then adjusted, as needed (+/- 200 kcals). Social and environmental barriers to adherence are also addressed, and problem-solving strategies are employed to overcome identified obstacles. Patients receive positive reinforcement throughout the process and previous visit notes are reviewed by the coach to personalize the discussion.

Training

Interventionists. All dietitians will receive 16-hours of standardized training in social cognitive strategy and motivational interview training for diet and physical activity behavior change and

will attend 1 half-day training session per year. In addition, interventionists will be provided with a training covering areas including overview of the LEAP study, overview of the LEAP lifestyle program, interviewing techniques, compliance and handling of private health information, team communication, ACCESS database tracking system, data entry and scheduling. Intervention-specific trainings will cover protocols related to each program goal (weight, improving nutrition, increasing physical activity, and managing stress), conducting sessions (sequence and content, scheduling, session reminders and call attempts), key principles underlying the intervention, retention, quality assurance, intervention fidelity and intervention evaluation. We will emphasize culturally sensitive delivery and tailored recommendations to meet the needs of the diverse patients.

Fidelity to the Intervention Protocol and Monitoring of the Intervention Process. Our systematic approach to intervention fidelity follows a framework of five key components: trial design, training, and intervention delivery, receipt, and enactment.[22]

Trial design includes intervention "dose," i.e., the standardized length, duration, and format of the virtual session with the lifestyle coach.

Training (see also above) will be standardized by simultaneously training all interventionists; recruiting staff with equivalent credentials; and using a treatment manual, case illustrations, and role play. All lifestyle coaches will receive 16 hours of standardized training in skill building, role playing, cognitive strategy and motivational interviewing for reducing barriers and enhancing awareness of the benefits of a healthy diet and exercise, prior to initiating the intervention. Training in culturally sensitive delivery and tailored exercise and diet recommendations will be emphasized to meet the needs of the diverse patients in this study. Upon completion of the training session, coaches will receive a certification.

Intervention delivery will be standardized. Interventionists' adherence to treatment delivery, including motivational interviewing techniques. After receiving patient permission, interventionists will digitally record all one on one sessions. The dietitians will use digital recording devices and will record all of their telephone calls. The digital recorder creates electronic audio files that can be saved on a computer and later be selected for review by the project manager and/or investigators; thus, the coaches will not know which of their sessions will be reviewed for quality control purposes. The project manager will review a random sample of 10% of introductory calls and 10% of Step 3 calls.

Adherence will be qualitatively assessed as well as quantified using a checklist of process and content variables, including MI techniques. Feedback will be provided weekly, allowing protocol deviations, "difficult" contacts, and MI skills to be addressed quickly.

Intervention receipt, Interventionists will document session attendance in the study tracking system.

Intervention enactment, i.e., performance of skills in daily life, will be assessed during each session through verbal self-report and completion of self-monitoring records. Interventionists will document process measures (e.g., goals; barriers; frequency of self-monitoring) in the tracking system.

Theoretical and Conceptual Framework Guiding the PA and Diet Intervention. Based on Bandura's social cognitive theory[23-25] and the Transtheoretical model,[26] the protocol for individual counseling will follow a step-wise, phased approach to behavior change focusing on key personal, social, and environmental mediators. These constructs have been the basis of previous research on adherence to and determinants of healthy diet and PA; thus, there is empirical support for their efficacy.[27-32] Personal factors include: a) Stage of change, i.e., readiness to adopt behavior changes.[33-35] The intervention approach will be modified according to patients' current stage. b) Goal setting. Patients will be encouraged to set sequential, realistic, and short-term diet and PA goals. c) Self-efficacy, i.e., confidence in one's ability to adhere to healthy behaviors across situations, previously shown to predict dietary[32] and PA goal achievement.[36-38] The intervention is designed to enhance self-efficacy through successful experiences meeting short-term goals. d) Self-monitoring. Patients will be asked to self-monitor their diet and PA to increase awareness of lifestyle behaviors, progress, and barriers to meeting goals. e) Additional factors include positive outcome expectations and the physical benefits of healthy nutrition and PA.[29,32] Social factors include support for initiating healthy nutrition[39] and PA[29] from family and friends. Patients will be encouraged to incorporate their families into behavior change efforts; we will provide tips for seeking diet[40] and PA support[27] (e.g., asking a spouse to baby-sit in order to do PA). Environmental factors include cues for diet and PA behaviors. Barriers related to social and environmental factors will be specifically addressed in the curriculum. Stimulus-Control strategies include teaching patients to prompt themselves to engage in healthy behaviors. Problem-solving. This systematic approach includes defining barriers to behavior change and identifying solutions.[41] Relapse prevention strategies include defining differences between "slips" (i.e., a 2 - 3 day "step back") vs. "relapses" (i.e., abandoning efforts for 3 weeks). Dietitians will help patients identify "high risk" situations that can precipitate slips and relapses and problem solve ways to prevent them.

DATA COLLECTION (Table 1)

Table 1. Variables assessed by EMR or by survey

Variable	Pregnancy		Delivery	6 wks Postpartum‡	Source
	12 wks	33 wks			
**Primary outcome**					
Total GWG (IOM)			X		**EMR**
Rate of GWG	X	X	X		**EMR**
Birthweight/GA			X		**EMR**
Postpartum wt				X	**EMR**
Infant growth*			X	X	**EMR**
**Moderators**					
Age	X				**EMR**
Race/ethnicity	X				**Survey**
Parity	X				**Survey**
Education	X				**Survey**
Pre-pregnancy BMI	X*				**EMR**
**Mediators**					
Diet / PA changes	X	X			**Survey**
Social support	X	X			**Survey**
Self-weighing	X	X			**Survey**
**Patient Satisfaction**	X	X			**Survey**
**Intervention Only**					

# of uses of app	X	X	X		**Tool**
# of wts recorded	X	X	X		**Tool**
‡Postpartum; *assessed up to 12 months					

There are two sources of data used to evaluate this intervention: 1) electronic medical record (EMR) data, and 2) patient surveys.

EMR will be used to gather information at the regular clinic and laboratory visits during 10- and 32-week pregnancy assessments, at delivery and at 6 months and 12-months postpartum assessments. The EMR data also include information on pre-gravid weight, gestational weight gain, medical history, new pregnancy episodes, and perinatal complications.

Survey data will be collected from patients in the intervention group and in the standard care group by a 30-minute self-administered online survey and food frequency questionnaire at around 12 and 33 weeks of pregnancy. The survey will focus on demographic information, SES, medical history, behaviors (diet, physical activity), sleep, and adverse events.

Birthweight (grams), defined as (mean (SD) and centiles (large for gestational age (LGA) > 90th percentile, small for gestational age (SGA) < 10th, and LGA > 95th, SGA < 5th),[9] macrosomia (> 4,000 g) and low birthweight (< 2,500 g).

Infant growth trajectory (BMI z-score), assessed from birth to 12 months, will use weight and length obtained from the **EMR at birth**, 3-5 days, and 1, 2, 4, 6, and 12 months. Z-scores at birth and 6 and 12 months will be used in the models of infant growth.

Characteristics that may affect response and/or adherence to the intervention. We will collect data from patients that might affect response or adherence to the intervention. These include medical history; lifestyle variables such as sleep; demographics, SES; and social support for diet and PA. More specifically:

Medical History and Lifestyle Variables. Information on reproductive history, medical conditions, pregnancy weight gain, and pregnancy outcomes will be obtained from the EMR or the surveys. Pre-pregnancy weight will be obtained from the EMR.

Demographics and SES. Maternal race-ethnicity, education, income, marital status, number of people in the household and number of children in the household will be assessed at the baseline survey.

Measures related to Delivery and Receipt of the Intervention

Self-monitoring. We will obtain information on self-monitoring focusing on self-monitoring of weight, calorie and diet as well as activity from the smartphone app.

Data Safety and Monitoring Board (DSMB). We propose a DSMB to enhance the integrity of study procedures and data quality. We will conduct annual DSMB meetings that will involve board members as well as research scientists, programmers, and project managers. The meetings will involve a one-hour investigator-led presentation, corresponding to the contents outlined in a previously sent report two weeks in advance of the meeting. The contents of the presentation will include an overview of the study, the study aims and outcomes, study flow (e.g., recruitment and retention), intervention (e.g., retention, lifestyle coach fidelity, etc.), preliminary results and possible adverse events. Following the one-hour presentation, a closed session discussion between the board members will occur to provide them with an opportunity

to further discuss the study and issues presented and whether any should result in a pause to the study procedures, protocol changes, etc. A designated note-taker will be asked to take thorough minutes during the presentation and the closed session discussion after which s/he will disseminate them to all attendees of the meeting. The DSMB will receive annual reports and may request additional reports due to unforeseen problems. The occurrence of serious adverse events (AE) is not a large concern, but we will collect data on AEs from the EMR and study questionnaires.

The LEAP DSMB includes Dennis M. Black, Ph.D., biostatistician at the University of California, San Francisco, who serves as the chair of the DSMB; Wendy Bennett, M.D. MPH, internist and researcher at The Johns Hopkins University School of Medicine; and Naomi Stotland, M.D., obstetrician and researcher at the University of California, San Francisco.

Summary of Changes to the LEAP Protocol

OUTCOMES

Primary outcomes. In the original protocol, the primary outcomes included the proportion of women who meet the IOM GWG guidelines and the weekly rate of GWG. We added an additional primary outcome measure, total GWG.

Secondary outcomes. In the original protocol, the secondary outcomes were trimester-specific weekly rate of GWG and GWG trajectory throughout pregnancy. We added the following secondary outcomes: Change in moderate to vigorous physical activity (in MET hours/week); Overall diet quality according to the Healthy Eating Index-2015 (HEI-2015); Postpartum weight retention; The proportion of infants with appropriate birthweight; and Infant growth.

ADVERSE EVENTS

At the request of the Data and Safety Monitoring Board (DSMB), we added NICU admission as a new adverse event that we ascertained.

RECRUITMENT, ELIGIBILITY & EXCLUSION CRITERIA

- **Study Setting:**
  - In the original protocol, we planned to recruit from the San Francisco and Oakland medical centers. In our final protocol, we added Redwood City and Santa Clara.
- **Eligibility Criteria:**
  - **BMI.** In the original protocol, eligibility was based on BMI calculated using pre-pregnancy BMI in the one year before the last menstrual period. We later revised this to use the first prenatal weight, if available; if unavailable, we used the pre-pregnancy weight before the LMP.
  - **Gestational Age.** In the original protocol, any patient with a gestational age ≥ 8 weeks who has a measured weight is loaded into the Access tracking system for immediate recruitment. We revised the process so that if there is no measured weight, we wait until 10 gestational weeks to load patients into the tracking system. At 10 weeks, if there is still no measured weight, then we used the pre-pregnancy weight. We also added verbiage to the patient recruitment email asking them to complete Survey 1 before 15 gestational weeks.
- **Recruitment Modalities:**
  - Our initial protocol sent an initial recruitment email; our final protocol added a mailed recruitment letter, with the purpose of reaching eligible patients who don’t access their email.

RETENTION STRATEGIES

- **Retention strategies:**

    - Due to provider time burden, we were unable to conduct one-hour motivational interview training sessions as originally planned. We delivered the provider intervention entirely remotely via email.
    - Additional documents: We added a follow-up letter and email to intervention patients whom we were unable to reach.
- **Incentives:** In the original protocol, we aimed to give patients $40 for completion of two online surveys. In the final protocol, patients would receive up to one $60 Amazon gift card for completing Survey 1 and up to one $50 Amazon gift card for completing Survey 2.

INTERVENTION

Overview of Lifestyle Intervention

- In the final protocol, patients were offered different options for tracking diet (calories and fat intake), weight, and physical activity rather than those proposed in the original protocol. The options included:
    1) allowing patients to use the Fitbit smartphone application or the MyFitnessPal (MFP) application for tracking diet (the original protocol only allowed the MFP app)
    2) allowing patients to use their own Fitbit or Apple Watch, or to obtain a Fitbit from the study, rather than a Withings activity tracker watch in the original protocol
    3) having patients use a wireless BodyTrace ‘smart’ scale rather than a Nokia BodyPlus scale in the original protocol
- In the original protocol, we aimed to develop a personalized mobile health website that uses artificial intelligence to extract real-time weight, physical activity, and diet data; in the final protocol, we developed a personalized mobile health smartphone application for iPhone and Android users.
- In the original protocol, intervention patients were to receive automated text messages as part of the mobile health application; we use automated push alerts instead.
- Additional documents added: The lifestyle coaches use the WebMD Portion Control Guide for reference as needed with intervention patients.
- In the final protocol of the introductory intervention call where the lifestyle coach meets with a newly enrolled intervention patient, the coach ascertains whether the patient wants to update her physical activity goal based on activity during the first week of the intervention. The final intervention quality control checklist for the introductory call was updated to include this item.
- Similarly, in the final protocol of the biweekly Step 3 intervention calls where the lifestyle coach meets with an intervention patient, the coach ascertains progress made in reaching the physical activity goal. The final intervention quality control checklist for the Step 3 call was updated to include this item.

DATA COLLECTION

Overview of data collection

- **Survey 1.** In the final survey, we removed questions on birth weights of previous babies, and how much weight the patient wants to gain.

- **Surveys 1 and 2.** To minimize patient burden, we eliminated questions regarding social support that were in early versions of the surveys.
- **Survey 2.** We added questions regarding COVID-19 vaccination after vaccines became available. To minimize patient burden, we eliminated questions regarding the impact of the COVID-19 pandemic on the patient.
- **Delivery Survey.** One eligibility question for all patients is if they plan to stay in the geographic area and continue to receive care through delivery at the Kaiser Permanente Northern California health system. All patients are required to answer YES to this question in order to participate. However, a small number of patients did end up leaving the health system, so we implemented a delivery survey to send to them to ascertain delivery date, weight at delivery (pounds), whether they delivered a liveborn baby, and if so, their baby's sex and weight at delivery.

STATISTICAL ANALYSIS – SAMPLE SIZE AND POWER ANALYSES

Below are the power analyses from the original proposal. This plan was revised in 2023 as requested by the DSMB based on expected patient recruitment at that time. The revised sample size and power analyses are listed in the Statistical Analysis Plan at the end of this document.

This study will randomize 56 clinicians (randomization unit) and 2,040 patients (expected based on 2019 panel size) to the intervention and standard care groups. Given the group-randomization study design, calculation of minimum detectable treatment effects account for the expected intraclass correlation (ICC) of observations within clusters. The ICCs in patient-level outcomes of interest are expected to be quite small. Published estimates of within practice ICC for patient outcomes are generally less than 0.05 and are often between .001 and .02.[47-50] We therefore present minimum detectable effects across a reasonable range for the expected ICC (.001 - .05).[51] Relevant to the log binomial regression analysis of treatment arm differences in the proportion with GWG meeting IOM guidelines, we present minimum detectable risk ratios, intervention vs. standard care. Given preliminary data, we assume an expected 29% of patients will achieve appropriate total GWG in the standard care group. We have sufficient power (.80) to detect relative risks of 1.20 to 1.34 for expected range in ICC (two-sided test, α = .05).[52,53] In addition, the minimum detectable difference in mean rate of GWG ranges from 0.13 to 0.21 standard deviation (s.d.) units, or 0.021 to 0.034 kg/week, for expected range in ICC (assumed s.d. = 0.16 given preliminary data, 2-sided t-test, α = .05, power =.80).[52,53] These minimum detectable effects are of clinical significance and are not unreasonable to expect based on prior intervention research among pregnant individuals with overweight or obesity.[54-56]

SECTION 2

STATISTICAL ANALYSIS PLAN

Randomization. A covariate constrained randomization scheme is implemented to reduce the chance of between-arm imbalance on important covariates, including patient-level factors, by eliminating all allocations to the intervention and standard care arms that do not meet specified balance criteria from among all possible treatment allocations of the 56 clinicians at 4 medical facilities.[12] Covariates for balance include clinician age, patient race/ethnicity (Asian, Black, Hispanic, White, Other), patient body mass index (BMI) (overweight, obese), and number of eligibles (to help ensure balance on trial enrollment), all based on data in the one year prior to randomization. These variables are chosen given the expected association with study outcomes and/or intervention engagement and level of between cluster variability which would increase the likelihood of between-arm imbalance.

Primary outcomes. Log binomial regression will be used to estimate the population average mHealth intervention effect on meeting the IOM GWG guidelines with estimation via generalized estimating equations (GEE; marginal model), accounting for the within-clinician correlation among patients to obtain valid estimates of treatment effects and associated standard errors. The probability of appropriate total GWG will be modeled as a function of treatment arm and covariates used in the randomization procedure: clinician age, patient race/ethnicity, and patient BMI. In addition, this a priori specified set of model covariates will include patient age and parity in the regression models given their strong association with GWG. Linear regression will be used to provide point and interval estimates of the between-arm difference in mean rate of GWG.

Secondary Outcomes. The approach to the analyses of trimester-specific GWG (kg/week) will parallel that described above for the total rate of GWG. Linear mixed effects models will be used in analyses of the intervention in relation to GWG trajectory, accounting for the within-person correlation among repeated measurements (not necessarily equal numbers per subject) and within-clinician correlation between patients, to obtain valid estimates of treatment effects and associated standard errors. We will also use latent trajectory class modeling to identify and categorize patients with respect to patterns of GWG during pregnancy. The approach to the analyses of postpartum weight retention at 6 weeks, change in moderate to vigorous physical activity (12 weeks to 33 weeks; MET hrs/week), overall diet quality at 12 weeks (Healthy Eating Index-2015), and birthweight (all continuous), and proportion of infants with appropriate size for gestational age (binary) will parallel that described for the primary outcomes or rate of GWG and meeting the IOM GWG guidelines. The approach to the analyses of the intervention in relation to 12-month infant growth (BMI Z-score) trajectory will parallel that described for analyses of GWG trajectory.

Revised Power and Sample Size Requested by DSMB Based on Recruitment Rate.
With 58 clinicians and an expected 1,180 total recruited, the minimum detectable RRs for proportion meeting IOM, intervention vs. usual care, are 1.27, 1.29 and 1.37 for expected intraclass correlations of .001, .01, and .05 (expected proportion meeting IOM in usual care = .29, power =.80, alpha = .05). The calculations for minimum detectable difference in mean rate GWG remain the same: the minimum detectable differences in mean rate of GWG are 0.16, 0.18, 0.23 s.d. units across the range in expected ICC.

REFERENCES

1. QuickStats: Gestational Weight Gain* Among Women with Full-Term, Singleton Births, Compared with Recommendations - 48 States and the District of Columbia, 2015. *MMWR Morb Mortal Wkly Rep.* 2016;65(40):1121.
2. Azar KMJ, Bennett GG, Nolting LA, Rosas LG, Burke LE, Ma J. A framework for examining the function of digital health technologies for weight management. *Transl Behav Med.* 2018;8(2):280-294.
3. Goldstein RF, Abell SK, Ranasinha S, et al. Association of Gestational Weight Gain With Maternal and Infant Outcomes: A Systematic Review and Meta-analysis. *JAMA.* 2017;317(21):2207-2225.
4. Vesco KK, Sharma AJ, Dietz PM, et al. Newborn size among obese women with weight gain outside the 2009 Institute of Medicine recommendation. *ObstetGynecol.* 2011;117(4):812-818.
5. In: Rasmussen KM, Yaktine AL, eds. *Weight Gain During Pregnancy: Reexamining the Guidelines.* Washington (DC)2009.
6. Margerison Zilko CE, Rehkopf D, Abrams B. Association of maternal gestational weight gain with short- and long-term maternal and child health outcomes. *Am J Obstet Gynecol.* 2010;202(6):574 e571-578.
7. Sridhar SB, Darbinian J, Ehrlich SF, et al. Maternal gestational weight gain and offspring risk for childhood overweight or obesity. *Am J Obstet Gynecol.* 2014;211(3):259-258.
8. Rasmussen KM, Yaktine AL. In: *Weight Gain During Pregnancy: Reexamining the Guidelines.* Washington (DC)2009.
9. Aris IM, Kleinman KP, Belfort MB, Kaimal A, Oken E. A 2017 US Reference for Singleton Birth Weight Percentiles Using Obstetric Estimates of Gestation. *Pediatrics.* 2019;144(1).
10. Schauberger CW, Rooney BL, Brimer LM. Factors that influence weight loss in the puerperium. *Obstet Gynecol.* 1992;79(3):424-429.
11. Ohlin A, Rossner S. Maternal body weight development after pregnancy. *Int J Obes.* 1990;14(2):159-173.
12. Hayes RJ, Moulton LH. *Cluster Randomzed Trial.* Boca Raton: Chapman & Hall/CRC; 2009.
13. Diabetes Prevention Program (DPP) Research Group. The Diabetes Prevention Program (DPP): Description of lifestyle intervention. *Diabetes Care.* 2002;25(12):2165-2171.
14. Ferrara A, Hedderson MM, Albright CL, et al. A pragmatic cluster randomized clinical trial of diabetes prevention strategies for women with gestational diabetes: design and rationale of the Gestational Diabetes' Effects on Moms (GEM) study. *BMC Pregnancy Childbirth.* 2014;14:21.
15. Ferrara A, Hedderson MM, Brown SD, et al. The Comparative Effectiveness of Diabetes Prevention Strategies to Reduce Postpartum Weight Retention in Women With Gestational Diabetes Mellitus: The Gestational Diabetes' Effects on Moms (GEM) Cluster Randomized Controlled Trial. *Diabetes Care.* 2016;39(1):65-74.
16. Bandura A. *Social Foundations of Thought and Action: A social cognitive theory.* Vol 1st Edition. Englewood Cliffs, N.J: Prentice Hall; 1986.
17. Bandura A. *Self-efficacy: The exercise of control.* New York: W.H.Freeman; 1997.
18. Prochaska JO, DiClemente CC. Common processes of self-change in smoking, weight control and psychological distress. In: Shiffman S, Wills T, eds. *Coping and Substance Use.* New York: Academic Press; 1985:345-363.
19. The Diabetes Prevention Program (DPP): description of lifestyle intervention. *Diabetes Care.* 2002;25(12):2165-2171.
20. Brown SD, Hedderson MM, Ehrlich SF, et al. Gestational weight gain and optimal wellness (GLOW): rationale and methods for a randomized controlled trial of a lifestyle intervention among pregnant women with overweight or obesity. *BMC Pregnancy Childbirth.* 2019;19(1):145.
21. Bauer P, CSCS, ACSM-EP, ACSM-EIM. Pregnancy Physical Activity. In: Medicine ACoS, ed2020.
22. Borrelli B, Sepinwall D, Ernst D, et al. A new tool to assess treatment fidelity and evaluation of treatment fidelity across 10 years of health behavior research. *J Consult Clin Psychol.* 2005;73(5):852-860.
23. A B. Social Foundations of Thought and Action: A social cognitive theory. *Englewood Cliffs, NJ: Prentice Hall.* 1986(1st Edition ed).
24. A B. Social learning theory. *Englewood Cliffs, NJ: Prentice Hall.* 1977.
25. A B. Self-efficacy : the exercise of control. *New York: WHFreeman.* 1997.

26. Prochaska JO DC. Common processes of self-change in smoking, weight control and psychological distress. *In: Shiffman S, Wills T, eds Coping and Substance Use New York: Academic Press.* 1985:345-363.
27. Miller YD TS, Brown WJ. Mediators of physical activity behavior change among women with young children. *Am J Prev Med* 2002;;23:98-103.
28. Dishman RK, Buckworth J. Increasing physical activity: a quantitative synthesis. *Med Sci Sports Exerc.* 1996;28(6):706-719.
29. Dishman RK SJF. Determinants and interventions for physical activity and exercise. *In: Bouchard C, Shephard RJ, Stephens T, eds Physical activity, fitness, and health: International proceedings and consensus statement Champaign, IL: Human Kinetics Publishers.* 1994:214-238.
30. King AC, Blair SN, Bild DE, et al. Determinants of physical activity and interventions in adults. *Med Sci Sports Exerc.* 1992;24(6 Suppl):S221-236.
31. Jordan KC F-GJ, Klohe-Lehman DM et al. A nutrition and physical activity intervention promotes weight loss and enhances diet attitudes in low-income mothers of young children. *Nutr Res* 2008;;28:13-20.
32. AbuSabha R AC. Review of self-efficacy and locus of control for nutrition- and health-related behavior. *J Am Diet Assoc.* 1997;;97:1122-1132.
33. Glanz K PR, Kristal AR et al. Stages of change in adopting healthy diets: fat, fiber, and correlates of nutrient intake. *Health Educ Q* 1994;;21:499-519.
34. Kristal AR GK, Curry SJ, Patterson RE. How can stages of change be best used in dietary interventions? . *J Am Diet Assoc* 1999;99:679-684.
35. Ory MG JP, Bazzarre T. The Behavior Change Consortium: setting the stage for a new century of health behavior-change research. *Health Educ Res* 2002 Oct; 17 (5):500-511.
36. E M. The role of efficacy cognitions in the prediction of exercise behavior in middle-aged adults. *J Behav Med* 1992;15:65-88.
37. McAuley E CK, Rudolph DL, Lox CL. Enhancing exercise adherence in middle-aged males and females. *Prev Med* 1994;23:498-506.
38. Eyler AA M-KD, Rohm YD et al. Quantitative study of correlates of physical activity in women from diverse racial/ethnic groups: Women's Cardiovascular Health Network Project--introduction and methodology. *Am J Prev Med* 2003;25:5-14.
39. Wing RR JR. Benefits of recruiting participants with friends and increasing social support for weight loss and maintenance. *J Consult Clin Psychol* 1999;67:132-138.
40. ES P. Enhancing social support in weight loss management groups. *J Am Diet Assoc* 1993;93:1152-1156.
41. Perri MG NA, McKelvey WF, Shermer RL, Renjilian DA, Viegener BJ. Relapse prevention training and problem-solving therapy in the long-term management of obesity. *J Consult Clin Psychol* 2001;;69:722-726.
42. Oka RK KA, Young DR. Sources of social support as predictors of exercise adherence in women and men ages 50 to 65 years. *Womens Health* 1995;1(2):161-175.
43. Sallis JF PR, Grossman RM, et al. The development of self-efficacy scales for health related diet and exercise behaviors. *Health Educ Res* 1988;3:283-292.
44. Ball K, Crawford D. An investigation of psychological, social and environmental correlates of obesity and weight gain in young women. *Int J Obes (Lond).* 2006;30(8):1240-1249.
45. Kiernan M MS, Schoffman DE, Lee K, King AC, Taylor CB, Kiernan NE, Perri MG. Social support for healthy behaviors: scale psychometrics and prediction of weight loss among women in a behavioral program. *Obesity (Silver Spring)* 2012 20:756-764.
46. Cohen S MR, Kamarck T, Hoberman HM. Measuring the functional components of social support. *In: Sarason IG, Sarason SB, editors Social support: Theory, research and applications Boston, MA: Dordrecht.* 1985:73-94.
47. Adams G, Gulliford MC, Ukoumunne OC, Eldridge S, Chinn S, Campbell MJ. Patterns of intra-cluster correlation from primary care research to inform study design and analysis. *JClinEpidemiol.* 2004;57(8):785-794.
48. Littenberg B, MacLean CD. Intra-cluster correlation coefficients in adults with diabetes in primary care practices: the Vermont Diabetes Information System field survey. *BMCMedResMethodol.* 2006;6:20.

49. Parker DR, Evangelou E, Eaton CB. Intraclass correlation coefficients for cluster randomized trials in primary care: the cholesterol education and research trial (CEART). *ContempClinTrials.* 2005;26(2):260-267.
50. Smeeth L, Ng ES. Intraclass correlation coefficients for cluster randomized trials in primary care: data from the MRC Trial of the Assessment and Management of Older People in the Community. *Control ClinTrials.* 2002;23(4):409-421.
51. Donner AaK, N. *Design and Analysis of Cluster Randomization Trials in Health Research.* Wiley; 2010.
52. Donner A, Klar N. *Design and Analysis of Cluster Randomization Trials in Health Research.* Arnold; 2000.
53. *PASS 2021 Power Analysis and Sample Size Software* [computer program]. NCSS, LLC.; 2021.
54. Ferrara A, Hedderson MM, Brown SD, et al. A telehealth lifestyle intervention to reduce excess gestational weight gain in pregnant women with overweight or obesity (GLOW): a randomised, parallel-group, controlled trial. *Lancet Diabetes Endocrinol.* 2020;8(6):490-500.
55. Peaceman AM, Clifton RG, Phelan S, et al. Lifestyle Interventions Limit Gestational Weight Gain in Women with Overweight or Obesity: LIFE-Moms Prospective Meta-Analysis. *Obesity (Silver Spring).* 2018;26(9):1396-1404.
56. Herring SJ, Cruice JF, Bennett GG, Rose MZ, Davey A, Foster GD. Preventing excessive gestational weight gain among African American women: A randomized clinical trial. *Obesity (Silver Spring).* 2015.
