# Supplementary material for: Adaptive Mobile Health Intervention to Reduce Excess Gestational Weight Gain: A Cluster-Randomized Clinical Trial
Source: JAMA Netw Open. 2026 Apr 20;9(4):e268007. doi: 10.1001/jamanetworkopen.2026.8007 (PMC13096985; doi:10.1001/jamanetworkopen.2026.8007)
Supplement: Supplement 2. — eTable 1. Characteristics of patients who enrolled vs. did not enroll patients in the LEAP program eTable 2. Characteristics of enrolled trial patients who agreed versus did not agree to participate in the LEAP intervention eTable 3. Mean change in diet from baseline to follow up with mean differences by treatment condition eTable 4. Mean change in physical activity from baseline to follow up with mean differences by treatment condition eFigure 1. Patient reported self-weighing from baseline to follow up, by treatment condition [file jamanetwopen-e268007-s002.pdf]

Supplemental Online Content

Hedderson MM, Brown SD, Quesenberry CP, et al. Adaptive mobile health intervention to reduce excess gestational weight gain: a cluster-randomized clinical trial. *JAMA Netw Open*. 2026;9(4):e268007. doi:10.1001/jamanetworkopen.2026.8007

eTable 1. Characteristics of patients who enrolled vs. did not enroll patients in the LEAP program.
eTable 2. Characteristics of enrolled trial patients who agreed versus did not agree to participate in the LEAP intervention.
eTable 3. Mean change in diet from baseline to follow up with mean differences by treatment condition
eTable 4. Mean change in physical activity from baseline to follow up with mean differences by treatment condition
eFigure 1. Patient reported self-weighing from baseline to follow up, by treatment condition

This supplemental material has been provided by the authors to give readers additional information about their work.

© 2026 Hedderson MM et al. *JAMA Network Open*.

e.Table1. Characteristics of patients who enrolled vs. did not enroll patients in the LEAP program.

Patient Characteristics	Enrolled (n=1,284)	Not enrolled (n=1,842)
Age at pregnancy, years		
Mean (SD)	33.3 (4.6)	32.7 (5.0)
21-29	270 (21.0)	474 (25.7)
30-34	487 (37.9)	692 (37.6)
35-39	428 (33.3)	504 (27.4)
40-47	99 (7.7)	172 (9.3)
Pre-pregnancy BMI, kg/m²		
Mean (SD)	29.8 (3.8)	30.2 (3.9)
25.0-29.9	757 (59.0)	1022 (55.5)
30.0-39.9	527 (41.0)	820 (44.5)
Race/Ethnicity		
White	433 (33.7)	468 (25.4)
Black	84 (6.5)	154 (8.4)
Asian/Pacific Islander	308 (24.0)	462 (25.1)
Hispanic	329 (25.6)	583 (31.7)
Other	130 (10.1)	175 (9.5)
Neighborhood Deprivation Index (NDI)**		
Least deprived	467 (36.4)	617 (33.5)
Second quartile	372 (29.0)	509 (27.6)
Third quartile	258 (20.1)	373 (20.3)
Most deprived	182 (14.2)	336 (18.2)
Missing/Unknown	5 (0.4)	7 (0.4)

© 2026 Hedderson MM et al. *JAMA Network Open*.

e.Table 2. Characteristics of enrolled trial patients who agreed versus did not agree to participate in the LEAP intervention.

Patient Characteristics	Agreed to the intervention (n=381)	Did not agree to the intervention (n=270)
Age at enrollment, years		
Mean (SD)	33.6 (4.6)	33.2 (4.6)
21-29	73 (19.2)	60 (22.2)
30-34	140 (36.8)	101 (37.4)
35-39	128 (33.6)	93 (34.4)
40-46	40 (10.5)	16 (5.9)
Pre-pregnancy BMI, kg/m²		
Mean (SD)	29.8 (3.7)	29.4 (3.8)
25.0-29.9	223 (58.5)	175 (64.8)
30.0-39.9	158 (41.5)	95 (35.2)
Race/Ethnicity		
White	137 (36.0)	88 (32.6)
Black	23 (6.0)	13 (4.8)
Asian/Pacific Islander	92 (24.2)	68 (25.2)
Hispanic	88 (23.1)	75 (27.8)
Other	41 (10.8)	26 (9.6)
Education		
High school or less	19 (5.0)	25 (9.3)
Some college	74 (19.4)	62 (23.1)
College	123 (32.3)	90 (33.5)
Postgraduate	165 (43.3)	92 (34.2)
Missing/Unknown	0 (0.0)	1 (0.4)
Neighborhood Deprivation Index (NDI)**		
Least deprived	163 (43.0)	109 (40.7)
Second quartile	111 (29.3)	67 (25.0)
Third quartile	61 (16.1)	53 (19.8)
Most deprived	44 (11.6)	39 (14.6)
Missing/Unknown	2 (0.5)	2 (0.7)

© 2026 Hedderson MM et al. *JAMA Network Open*.

eTable 1. Mean change in diet from baseline to follow up with mean differences by treatment condition

	**Standard Care (n=530)**	**Intervention (n=591)**	**Between-condition difference in means* (95% CI)**
**AHEI-P[1] score**			
Baseline	42.1 (11.6)	41.9 (11.4)	-
Follow-up	43.2 (12.0)	42.7 (11.5)	-
Change	1.1 (10.0)	0.9 (10.0)	-0.52 (-1.67 to 0.62)
**HEI 2015[2] score**			
Baseline	64.3 (9.2)	64.5 (9.4)	-
Follow-up	64.0 (9.1)	64.1 (9.9)	-
Change	-0.3 (8.4)	-0.4 (8.9)	-0.12 (-1.04 to 0.81)
**Total calories per day, kCal**			
Baseline	1380.1 (631.8)	1331.4 (583.1)	-
Follow-up	1411.6 (657.4)	1372.5 (595.7)	-
Change	31.5 (546.8)	41.1 (527.8)	-8.19 (-70.60 to 54.22)
**Percent of calories from total fat**			
Baseline	38.8 (6.4)	39.0 (7.0)	-
Follow-up	40.1 (5.9)	39.8 (6.4)	-
Change	1.3 (6.7)	0.7 (6.9)	-0.49 (-1.19 to 0.20)
**Percent of calories from saturated fat**			
Baseline	12.7 (2.7)	12.9 (2.9)	-
Follow-up	13.4 (2.7)	13.4 (2.9)	-
Change	0.7 (2.8)	0.5 (2.9)	-0.11 (-0.39 to 0.18)

Analyses included all eligible consented participants who remained pregnant and were sent the follow-up survey at 33 weeks (standard care group, n=530; intervention group, n=591). Baseline was assessed at 10 [IQR: 9-12] weeks' gestation, and follow-up at 33 [IQR:33-35] weeks' gestation.

* All models were specified using the standard care group as the reference and were adjusted for pre-pregnancy BMI, race/ethnicity, clinician facility, clinician age, maternal age, parity, the respective baseline value, and the time interval between surveys. Mean differences were estimated using linear regression with generalized estimating equations (GEE) to account for within-clinician correlation.

[1.] The Alternative Healthy Eating Index-Pregnancy (AHEI-P) is an adapted version of the Healthy Eating Index (HEI) that excludes alcohol and adds calcium, folate, and iron to make it a suitable measure of dietary patterns in pregnant women. Scores are on a scale of 0 to 130.
[2.] The Healthy Eating Index (HEI) is a measure of dietary patterns that assess how well diet aligns with key dietary recommendations. Scores are on a scale of 0 to 100.

© 2026 Hedderson MM et al. *JAMA Network Open*.

eTable 2. Mean change in physical activity from baseline to follow up with mean differences by treatment condition

Type of activity, MET h per week	Standard Care (n=530)†	Intervention (n=591)†	Between-condition difference in means* (95% CI)
**Total energy expenditure**			
Baseline	139.5 (97.4)	132.5 (100.1)	-
Follow-up	136.0 (96.3)	131.2 (101.8)	-
Change	-3.5 (88.1)	-1.3 (93.2)	0.22 (-9.12 to 9.55)
**Sedentary behavior**			
Baseline	57.0 (36.9)	59.0 (37.8)	-
Follow-up	55.5 (36.3)	56.5 (37.0)	-
Change	-1.5 (37.4)	-2.4 (37.3)	-0.03 (-3.36 to 3.30)
**Light activity**			
Baseline	73.7 (48.1)	68.5 (49.0)	-
Follow-up	75.2 (47.7)	71.2 (49.5)	-
Change	1.5 (43.4)	2.7 (42.0)	-0.12 (-4.34 to 4.11)
**Moderate activity**			
Baseline	64.7 (63.5)	62.8 (64.2)	-
Follow-up	60.1 (61.1)	59.6 (65.5)	-
Change	-4.7 (59.8)	-3.2 (65.7)	0.73 (-5.65 to 7.10)
**Vigorous activity**			
Baseline	2.1 (4.3)	2.1 (4.0)	-
Follow-up	1.3 (3.4)	1.2 (2.6)	-
Change	-0.8 (4.2)	-1.0 (3.9)	-0.18 (-0.49 to 0.13)
**Household activity**			
Baseline	88.4 (72.5)	78.3 (67.3)	-
Follow-up	83.6 (65.7)	80.2 (68.5)	-
Change	-4.8 (52.8)	1.9 (53.3)	3.65 (-1.92 to 9.21)
**Transport physical activity**			
Baseline	10.4 (17.5)	11.0 (19.0)	-
Follow-up	14.8 (23.6)	13.8 (21.9)	-
Change	4.4 (23.1)	2.8 (24.0)	-1.19 (-3.76 to 1.38)
**Non-vigorous sports**			
Baseline	11.1 (12.4)	10.9 (12.2)	-
Follow-up	9.2 (11.5)	9.1 (9.5)	-
Change	-2.0 (12.5)	-1.8 (12.4)	-0.22 (-1.34 to 0.90)
**Moderate and vigorous sports activity**			
Baseline	69.0 (74.6)	67.5 (77.2)	-
Follow-up	63.7 (74.7)	63.6 (79.0)	-
Change	-5.3 (71.3)	-3.9 (78.9)	0.80 (-6.90 to 8.51)

Analyses included all eligible consented participants who remained pregnant and were sent the follow-up survey at 33 weeks (standard care group, n=530; intervention group, n=591). Baseline was assessed at 10 [IQR: 9-12] weeks' gestation, and follow-up at 33 [IQR:33-35] weeks' gestation.

© 2026 Hedderson MM et al. *JAMA Network Open*.

* All models were specified using the standard care group as the reference and were adjusted for pre-pregnancy BMI, race/ethnicity, clinician facility, clinician age, maternal age, parity, the respective baseline value, and the time interval between surveys. Mean differences were estimated using linear regression with generalized estimating equations (GEE) to account for within-clinician correlation.

.

eFigure 1. Patient reported self-weighing from baseline to follow up, by treatment condition

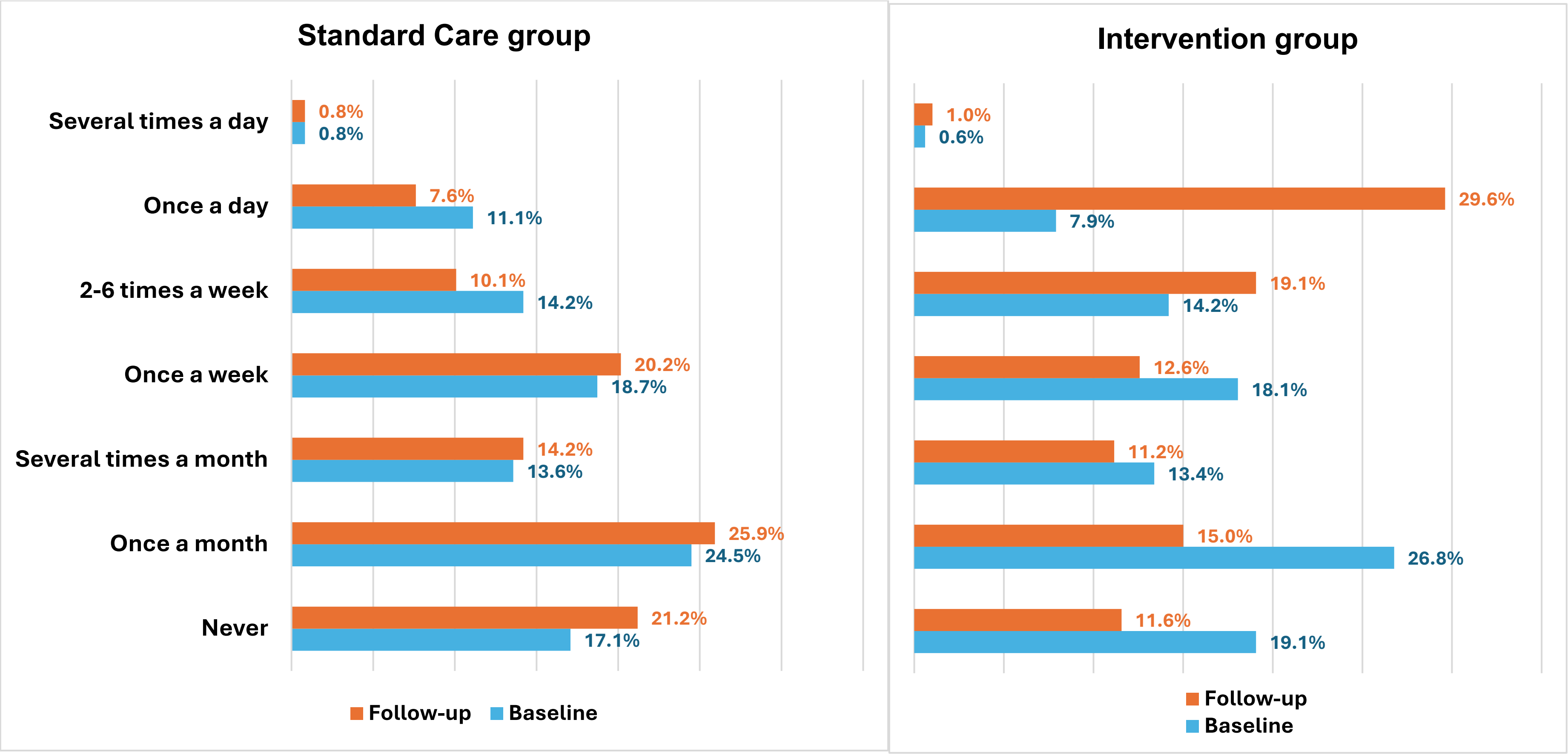

* Calculated among 493 patients in the intervention group and 486 patients in the standard care group who reported self-weighing frequency at both baseline (10 [IQR: 9-12] weeks' ges

© 2026 Hedderson MM et al. *JAMA Network Open*.
