# Supplementary material for: Adaptive Mobile Health Intervention to Reduce Excess Gestational Weight Gain: A Cluster-Randomized Clinical Trial
Source: JAMA Netw Open. 2026 Apr 20;9(4):e268007. doi: 10.1001/jamanetworkopen.2026.8007 (PMC13096985; doi:10.1001/jamanetworkopen.2026.8007)
Supplement: Supplement 4. — Data Sharing Statement [file jamanetwopen-e268007-s004.pdf]

Data Sharing Statement

Hedderson. Adaptive Mobile Health Intervention to Reduce Excess Gestational Weight Gain. *JAMA Netw Open*. Published April 20, 2026. doi:10.1001/jamanetworkopen.2026.8007

Data
Additional Information: ClinicalTrials.govNCT03880461
Data available: Yes
Data types: Deidentified participant data, Data dictionary
How to access data: request consideration for access to data, please contact, Project Manager, via email: Sneha.x.sridhar@kp.org
When available: beginning date: 08-21-2026

Supporting Documents
Document types: None

Additional Information
Who can access the data: Researchers whose proposed use of the data has been approved.
Types of analyses: For pre-planned analyses and meta-analyses (if applicable)
Mechanisms of data availability: After approval of proposal
